# Screening positive rate and radiographically confirmed prevalence of scoliosis in Chinese children and adolescents from 2020 to 2025: a systematic review and meta-analysis

**DOI:** 10.3389/fped.2026.1954010

**Published:** 2026-10-30

**Authors:** Shiyi Lu, Zan Huang, Youzhen Zhang, Zhikai Qin, Wei Yang, Yuying Wu, Shuying Fan, Wenxin Xu

**Affiliations:** 1 School of Physical Education and Sports Science, Fujian Normal University, Fuzhou, China; 2 Post-Doctoral Station of Sport Science, Fujian Normal University, Fuzhou, China

**Keywords:** children and adolescents, China, meta-analysis, scoliosis, screening

## Abstract

**Background:**

School-based scoliosis studies often report screening positivity and radiographic confirmation as equivalent prevalence measures despite differences in diagnostic stage and denominator. In this study, we separately estimated screening-positive rates and radiographically confirmed prevalence among Chinese children and adolescents surveyed during 2020–2025.

**Methods:**

Six databases were searched for Chinese- or English-language studies published from January 2020 to May 2026. Eligible school- or population-based cross-sectional studies included participants aged 6–18 years or primary and secondary school students. Random-effects meta-analyses were performed using logit-transformed proportions and restricted maximum likelihood estimation. Heterogeneity was assessed using *I*^2^, *τ*^2^, and 95% prediction intervals (PIs). The review was registered in PROSPERO (CRD420261443909).

**Results:**

A total of 103 independent studies were included. Eighty-four studies contributed 95 screening-positive estimates from 6,045,953 participants, yielding a pooled screening-positive rate of 3.43% (95% CI: 2.98%–3.93%; *I*^2^ = 99.84%; 95% PI: 0.86%–12.70%). Twenty-three studies involving 1,162,720 participants yielded a radiographically confirmed prevalence rate of 1.83% (95% CI: 1.17%–2.86%; *I*^2^ = 99.85%; 95% PI: 0.18%–16.41%). Female-to-male prevalence ratios were 1.58 and 1.49, respectively. Geographic region was associated with radiographically confirmed prevalence at the study level (*P* = 0.011).

**Conclusions:**

Both outcomes showed extreme heterogeneity and wide prediction intervals. The pooled estimates should therefore be interpreted as random-effects averages across heterogeneous study populations rather than fixed national prevalence values. Screening-positive rate and radiographically confirmed prevalence should not be treated as equivalent measures, and regional meta-regression findings should be regarded only as exploratory.

**Systematic Review Registration:**

https://www.crd.york.ac.uk/PROSPERO/, identifier PROSPERO CRD420261443909.

## 1 Introduction

Scoliosis is a common three-dimensional spinal morphological abnormality that occurs during the growth and development period of children and adolescents, which may affect appearance, mobility, mental health, and the spinal function in adulthood (1, 2). School screening can identify suspected cases before obvious symptoms appear and provide a basis for re-examination, imaging confirmation, and follow-up (2, 3).

The school screening pathways are not uniform: postural observation, Adams forward-bend test, trunk rotation angle (ATR), or national screening standards are used to identify suspected or high-risk individuals; standing full-spine X-ray and Cobb angle criteria are used to confirm structural scoliosis. The diagnostic stages and denominators of these two types of indicators are different, and being called “prevalence” in both cases will weaken the comparability between regions and countries (2–5).

A previous population-wide analysis in China reported a positive rate of initial screening of 3.97% and an imaging final screening prevalence rate of 1.20% (5); a recent global X-ray confirmation meta-analysis reported a prevalence of 1.65%, with a range of 0.05%–16.67% among different countries (6). Differences may be caused by study years, age, screening tools, thresholds, referral and follow-up procedures, and the definition of the denominator.

This study limits the actual investigation period to 2020–2025 to reduce differences associated with historical screening standards and imaging conditions and separately analyzes screening-positive rates and Cobb angle–confirmed prevalence as outcomes representing different stages of the screening and diagnostic pathway. The study also evaluates gender, educational stage, and urban–rural and regional differences and examines the robustness and heterogeneity of the results through prediction intervals, sensitivity analysis, three-level models, and meta-regression.

## 2 Methods

### 2.1 Research design and protocol registration

This systematic review was conducted and reported in accordance with the Preferred Reporting Items for Systematic Reviews and Meta-Analyses 2020 statement (PRISMA 2020) (7). The review was registered in PROSPERO (CRD420261443909). Prespecified subgroup analyses included school stage, urbanicity, geographic region, and screening framework, as specified in the PROSPERO record. Screening-positive rate and radiographically confirmed prevalence were treated as two separate outcomes because they represent different stages of the screening and diagnostic pathway and use different denominators; therefore, they were not combined into a single overall prevalence estimate.

### 2.2 Information sources and search strategy

Systematic searches were conducted in China National Knowledge Infrastructure (CNKI), Wanfang Data Knowledge Service Platform, PubMed, Embase, Scopus, and Web of Science Core Collection. The databases were searched for studies published from 1 January 2020 to 30 May 2026. Searches were restricted to publications in Chinese or English. The actual survey year was verified during full-text screening. In addition, the reference lists of included studies and relevant reviews were screened, forward citation searching was conducted, and authors or experts were contacted where necessary to identify additional potentially eligible studies.

The search formula consisted of the research subjects, scoliosis, screening/epidemiological outcomes, and four Chinese concept groups and was adjusted according to the subject terms and field syntax of each platform. The complete search formula in PubMed was: (“Child”[Mesh] OR child*[tiab] OR adolescen*[tiab] OR student*[tiab] OR schoolchild*[tiab] OR “primary school”[tiab] OR “middle school”[tiab] OR “secondary school”[tiab]) AND (“Scoliosis”[Mesh] OR scoliosis[tiab] OR “spinal curvature”[tiab] OR “spinal curvature abnormal*”[tiab] OR “spinal deform*”[tiab]) AND (“Mass Screening”[Mesh] OR screen*[tiab] OR prevalence[tiab] OR epidemiolog*[tiab] OR “detection rate”[tiab] OR “Adams forward bend test”[tiab] OR “angle of trunk rotation”[tiab] OR ATR[tiab] OR radiograph*[tiab] OR “Cobb angle”[tiab]) AND (“China”[Mesh] OR China[tiab] OR Chinese[tiab]). The complete and reproducible search formulas for other databases can be found in Supplementary Table S1.

### 2.3 Inclusion and exclusion criteria

The study must meet the following conditions: (1) It should be based on cross-sectional surveys, prevalence surveys, or spinal curvature screening studies conducted in schools or the general population; (2) The study subjects should be Chinese children and adolescents aged 6–18 years or school students, or the data of the target age range can be extracted independently; (3) The actual survey period was from 1 January 2020 to 31 December 2025, and eligible studies were published between 1 January 2020 and 30 May 2026; (4) It should use body posture examination, Adams flexion test, ATR measurement, national screening standards, or standing position spinal X-ray to evaluate spinal curvature; (5) The study should report both the number of positive cases and the total number of participants; if not directly reported, data that can be used for calculation should also be provided; (6) The full text in Chinese or English should be available. The time limit is from 2020 to 2025, aiming to reflect recent school screening practices, current standards and equipment conditions, and recent public health policy background and to reduce clinical and methodological heterogeneity caused by the mixture of historical screening methods and current methods.

The exclusion criteria include hospital-visited or treated populations, populations selected on the basis of known congenital, neuromuscular, syndromic, or other secondary causes of scoliosis, studies that cannot separate the data of the target age range, non-cross-sectional studies, case reports or reviews, studies that do not directly report the outcome of spinal curvature, and studies that cannot obtain the number of confirmed or screened confirmed cases and the total number. When the same survey population is published repeatedly, the version with the most complete sample coverage, the clearest outcome definition, or the most comprehensive method report should be retained first.

### 2.4 Literature screening and data extraction

All records underwent duplicate removal, title and abstract screening, and full-text eligibility assessment. Two reviewers independently conducted the screening and eligibility assessment. Any disagreements were resolved through discussion, with consultation of a third reviewer when necessary. All final inclusion and exclusion decisions were made by the review team.

The two researchers independently extracted data using a predesigned structured table, and the third researcher verified it. The extracted items included the first author, publication year, survey year, region, research design, sampling method, age, gender, educational stage, urban–rural attribute, sample size, response rate, screening tool, positive threshold, X-ray referral and re-examination process, Cobb angle diagnostic criteria, positive number, and denominator. For imaging outcomes, all the screened student numbers, the number of initially positive students, the referral numbers, the actual number of students who underwent X-ray examination, the number of confirmed cases, and the follow-up loss situations in each stage were further recorded, and based on this, it was determined whether the diagnostic path was complete.

For the additional analysis of study setting, information on the organization and source of the screening program was also extracted from the original reports. Studies explicitly conducted as routine student health surveillance, common-disease monitoring, or government/public-health screening programs were classified as routine/official public-health screening. Investigator-initiated local surveys were classified as investigator-initiated/local academic studies. Studies without sufficient information for a reliable classification were recorded as unclear. Sample size was not used to determine program setting.

### 2.5 Outcome definition and diagnostic levels

The screening-positive rate was defined as the number of students identified as having suspected scoliosis, being at high risk, or screening positive according to the criteria used in the original study divided by the total number of students screened. Screening procedures included postural examination, the Adams forward-bend test, ATR assessment, national screening standards, or combinations of these procedures.

The primary radiographic outcome, termed radiographically confirmed prevalence among all screened students, was defined as the number of students who met the radiographic Cobb angle criteria specified in the original study divided by the total number of students participating in the screening program. The proportion of confirmed cases among students who screened positive and the proportion of confirmed cases among students who actually underwent radiography were treated as separate pathway indicators. These measures are influenced by referral procedures and completion of radiographic follow-up and therefore do not represent prevalence in the entire screened population. Accordingly, they were not included in the primary prevalence analysis.

For descriptive subgroup analyses, screening-positive estimates were classified according to the principal screening framework reported in each study. Because postural examination, the Adams forward-bend test, and ATR measurement were frequently used in combination, these categories were not intended to represent mutually exclusive clinical procedures. Studies explicitly reporting implementation according to GB/T 16133-2014 were classified as national-standard screening. Among the remaining studies, estimates were classified according to the principal reported criterion used to define screening positivity, including clinical/postural screening or ATR-based screening. These study-level categories were used to describe variation in screening-positive rates and were not interpreted as direct comparisons of the diagnostic performance of different screening procedures. Radiographically confirmed outcomes based on Cobb angle assessment were analyzed separately using the total screened population as the denominator. A sensitivity analysis examined the effect of excluding studies with known incomplete radiographic follow-up.

The review focused on scoliosis identified in children and adolescents from school-based or general-population surveys, rather than populations selected on the basis of known congenital, neuromuscular, syndromic, or other secondary causes of scoliosis. When a report did not specify the etiological subtype of scoliosis, we retained the overall scoliosis outcome reported by the original authors rather than reclassifying it as adolescent idiopathic scoliosis. The available information on etiological subtypes was insufficient for separate pooling of idiopathic and non-idiopathic scoliosis.

When a study reported both an overall estimate and estimates stratified by sex, school stage, or urbanicity, only the overall estimate was included in the primary analysis, whereas stratified estimates were used only in the corresponding subgroup analyses. This approach avoided double-counting of the same participants.

For school stage, general senior secondary schools, vocational senior secondary schools, and higher vocational schools were grouped as senior secondary education. For urbanicity, urban districts, cities, towns, and urban schools were classified as urban, whereas rural areas, villages, rural schools, suburbs, outlying counties, county districts, and county-level areas were classified as non-urban.

Study locations were classified as eastern, central, or western China according to the national three-region classification. Liaoning, Jilin, and Heilongjiang were assigned to eastern China; Shanxi, Anhui, Jiangxi, Henan, Hubei, and Hunan were assigned to central China; and the western provinces and autonomous regions were assigned to western China.

### 2.6 Methodological quality assessment

The methodological quality of the included studies was assessed using the JBI Critical Appraisal Checklist for Studies Reporting Prevalence Data (8, 9). All nine items were evaluated using predefined operational criteria, which are provided in Supplementary Table S3, and each item was rated as Yes, No, Unclear, or Not applicable. For item 3, the absence of a reported *a priori* sample-size calculation was not, by itself, considered grounds for an Unclear rating; sample-size adequacy was judged according to the achieved sample size, study design and population coverage, and the adequacy of the sample for prevalence estimation. Two reviewers independently conducted the quality assessment, and any disagreements were resolved through discussion. Because the JBI does not specify a validated numerical score or uniform threshold for classifying overall study quality, no total score or overall quality category was assigned.

### 2.7 Statistical analysis

All statistical analyses were performed in Python 3.13.5 using NumPy 2.3.5, SciPy 1.17.0, statsmodels 0.14.6, and Matplotlib 3.10.8. Separate meta-analyses of single proportions were conducted for screening-positive rates and Cobb angle–confirmed prevalence. Study-specific proportions were logit-transformed and pooled using inverse-variance random-effects models, with between-study variance (*τ*^2^) estimated by restricted maximum likelihood (REML). Pooled estimates and their 95% confidence intervals (CIs) were back-transformed to the original proportion scale. A continuity correction of 0.5 was specified for zero-event or all-event proportions in the logit inverse-variance analysis. However, no effect size included in either primary analysis had zero events or 100% prevalence, and no continuity correction was therefore required. Alternative statistical approaches were examined in sensitivity analyses to assess the robustness of the pooled estimates. The Hartung–Knapp adjustment was used only in sensitivity analyses and not for primary inference (10).

Statistical heterogeneity was assessed using Cochran's Q, *I*^2^, and *τ*^2^, and 95% prediction intervals (PIs) were calculated to characterize the expected range of true prevalence estimates in comparable populations. Because prevalence meta-analyses frequently include very large studies with small within-study sampling errors, *I*^2^ may approach 100% even when the absolute magnitude of between-study variation is more informative. Therefore, heterogeneity was interpreted jointly using *τ*^2^, prediction intervals, subgroup analyses, meta-regression, and sensitivity analyses rather than *I*^2^ alone (11–16).

Subgroup analyses included diagnostic stage, sex, school stage, urbanicity, geographic region, and screening framework classification. For studies reporting data for both girls and boys, within-study prevalence ratios (PRs) comparing girls with boys were calculated. Similarly, for studies reporting both urban and non-urban populations, within-study PRs comparing urban with non-urban students were calculated. PRs were log-transformed and pooled using REML random-effects models. For study-level stratified variables such as school stage, geographic region, diagnostic stage, and screening framework classification, subgroup-specific pooled estimates were reported without calculating ratios between pooled estimates derived from different studies.

The primary analysis of radiographically confirmed prevalence included studies reporting Cobb angle–confirmed cases with the total screened population as the denominator. For these studies, information on referral for radiography, completion of radiographic examination, and loss to follow-up was extracted from the original reports where available. Because reporting of the radiographic pathway was incomplete across studies, absence of reported loss to follow-up was not interpreted as evidence of complete radiographic follow-up.

Sensitivity analyses included leave-one-study-out analysis, exclusion of studies with potential sample overlap or insufficient independence, aggregation of multiple survey years reported within the same article, application of strict age boundaries, exclusion of the largest or very large samples, exclusion of small samples, and exclusion of studies with explicitly documented incomplete radiographic follow-up. Pooled estimates were also re-estimated using alternative statistical approaches, including the Hartung–Knapp adjustment, DerSimonian–Laird estimation, Freeman–Tukey double-arcsine transformation, a binomial-logit generalized linear mixed model (GLMM), and a fixed-effect model. The GLMM was fitted directly to the study-level event counts and denominators using a binomial distribution and logit link, without continuity correction. The fixed-effect model was used only for methodological comparison and not for primary inference.

Because a small number of studies contributed more than one screening-positive estimate from different survey years or analytical samples, effect-size dependence was additionally examined using a three-level meta-analytic model. Effect sizes were nested within independent studies, allowing heterogeneity to be partitioned into within-study and between-study variance components. The variance components of the three-level model were estimated using restricted maximum likelihood (REML). The 95% confidence interval for the pooled three-level estimate was calculated on the logit scale using the normal approximation (*μ* ± 1.96 × SE) and then back-transformed to the proportion scale. The conventional two-level random-effects model was retained as the primary analysis because most independent studies contributed only one effect size, whereas the three-level model and an alternative analysis aggregating multiple estimates within the same article were used to assess the robustness of the results to effect-size dependence.

To examine whether the inverse association between sample size and screening positivity could reflect differences in screening program setting, an additional *post hoc* sensitivity analysis compared routine/official public-health screening programs with investigator-initiated/local academic studies. Studies with unclear program setting were excluded from this comparison. For studies reporting multiple screening-positive estimates, numerators and denominators were aggregated at the independent-study level. The same random-effects approach as in the primary analysis was used, and the between-setting difference was evaluated using a study-level moderator test.

Univariable and multivariable random-effects meta-regression analyses were used to explore potential sources of between-study heterogeneity. For studies contributing multiple screening-positive effect sizes, numerators and denominators were aggregated by independent study ID before meta-regression so that each study contributed one study-level observation. For screening positivity, the examined covariates included survey year, natural logarithm of sample size, geographic region, and screening framework classification. For radiographically confirmed prevalence, the examined covariates included survey year, natural logarithm of sample size, and geographic region. Radiographic completion was not entered into meta-regression because sufficiently comparable completion data were available for only a limited number of studies. Meta-regression findings were interpreted as study-level observational associations rather than individual-level causal effects (17, 18).

Influence diagnostics were conducted for the final multivariable meta-regression models using externally studentized residuals, Cook's distance, leverage, and DFBETAS. Studies identified as potentially influential were reviewed against the original reports, and leave-one-study-out analyses were used to evaluate their influence on the estimated regression coefficients and model conclusions.

Because meta-analyses of single proportions differ statistically from meta-analyses of comparative intervention effects, conventional funnel plots, Egger's test, and trim-and-fill methods may be affected by proportion boundary effects, the structural relationship between proportions and their standard errors, study precision, and substantial between-study heterogeneity. These methods were therefore not used for primary assessment of publication bias. Robustness was instead evaluated through the completeness of the search strategy, sensitivity analyses, and assessment of small-study effects (19–23).

## 3 Results

### 3.1 Literature screening

A total of 1,603 records were identified through database searching, including 37 from CNKI, 391 from Wanfang, 187 from PubMed, 396 from Embase, 391 from Scopus, and 201 from the Web of Science Core Collection. Before screening, 235 duplicate records were removed, leaving 1,368 records for title and abstract screening. Of these, 1,218 records were excluded, including studies with survey years outside the eligibility period (*n* = 148), reviews, guidelines, or study protocols (*n* = 267), studies unrelated to spinal disorders (*n* = 326), non-epidemiological or non-prevalence studies (*n* = 427), and studies without extractable prevalence or screening-positive data (*n* = 50). A total of 150 full-text articles were assessed for eligibility. Of these, 47 articles were excluded for the following reasons: ineligible study population (*n* = 12), ineligible age range (*n* = 18), inappropriate study design (*n* = 5), insufficient extractable data (*n* = 5), failure to report scoliosis or abnormal spinal curvature outcomes separately (*n* = 5), and overlapping study populations (*n* = 2). Ultimately, 103 independent studies were included in the systematic review and meta-analysis. The study selection process is presented in Figure 1.

**Figure 1:**
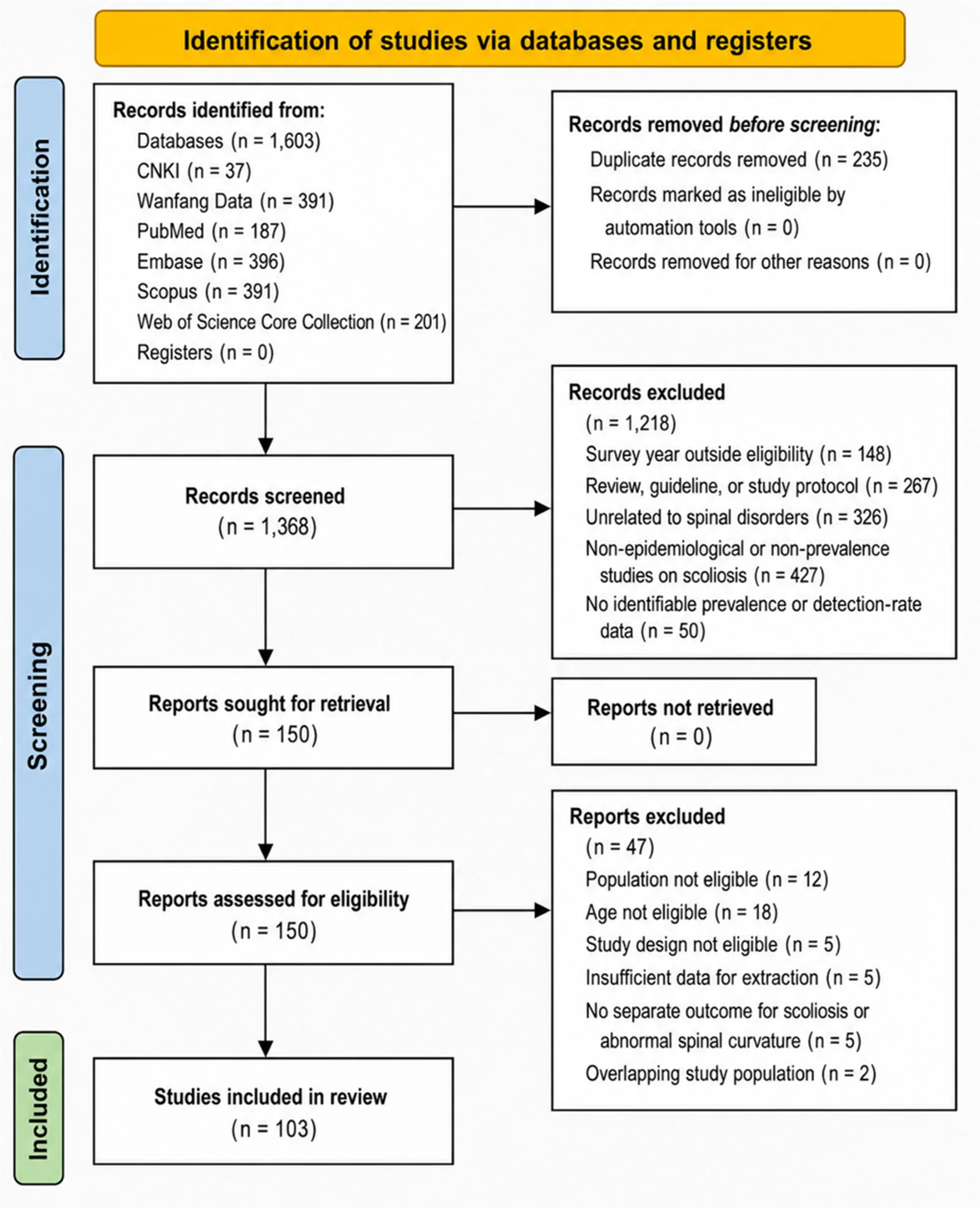
A flowchart of literature screening.

### 3.2 Characteristics of included studies

Detailed information on all included studies is provided in Supplementary Table S2. Study-specific screening procedures, positivity criteria or ATR thresholds, and Cobb angle diagnostic thresholds are presented in Supplementary Table S2. A total of 103 cross-sectional studies were included (24–126), with survey periods ranging from 2020 to 2025. Of these, 80 studies reported screening-positive outcomes only (24–44, 46–48, 50, 52, 56, 58, 61, 62, 64–66, 68–82, 84, 86–89, 91–98, 102–104, 107–115, 117, 119–121, 123–125), 19 reported Cobb angle–confirmed outcomes only (45, 51, 53–55, 57, 59, 63, 67, 83, 85, 90, 99–101, 106, 116, 122, 126), and four reported both outcomes (49, 60, 105, 118). Thus, the screening-positive analysis included 84 studies (80 screening-positive only and four reporting both outcomes), whereas the radiographically confirmed analysis included 23 studies (19 radiographic only and the same four overlapping studies), corresponding to 103 unique studies overall. The 84 studies contributing to the screening-positive analysis provided 95 effect sizes because some studies reported multiple survey years or stratified samples.

Among the 23 studies contributing to the radiographically confirmed prevalence analysis, 16 defined scoliosis using a Cobb angle threshold of ≥10°, whereas seven used >10°. Radiographic protocols and Cobb angle assessment procedures were not fully standardized across studies, and details regarding positioning, projection, and assessor procedures were inconsistently reported.

The included populations covered primary, junior secondary, and senior secondary school stages. Ninety studies included primary school students (24, 26–30, 32–48, 50–52, 54, 55, 57, 58, 60–63, 65, 67–71, 73, 75–96, 98–113, 115, 117–126), 63 included junior secondary school students (24, 26–28, 30–34, 40–54, 56, 58, 61–65, 67–69, 71–73, 75, 79, 90, 93, 98, 99, 101–104, 107–113, 117–125), and 41 included senior secondary school students (24–28, 30, 32, 34, 41–44, 47, 48, 50–52, 58, 59, 61, 62, 65, 66, 69, 74, 75, 97, 98, 104, 107–109, 111, 114, 116–118, 120, 121, 123, 125).

Screening components were frequently used in combination. The Adams forward-bend test was used in 92 studies (24–28, 30–58, 60–75, 77–82, 85, 87–101, 103–107, 109–113, 115–123, 125), ATR measurement was used in 84 studies (24–28, 30–32, 34–50, 52, 53, 55–62, 64–66, 68–75, 77–83, 87–98, 100, 103–107, 109–117, 119–125), and 14 studies explicitly reported screening according to GB/T 16133-2014 (74, 76, 78, 84, 86, 87, 92, 95, 96, 98, 102, 109, 119, 121). Counts for school stage and screening components were therefore not mutually exclusive.

By study location, 56 studies were conducted in eastern China (26, 29, 32, 35–37, 39, 40, 42–44, 46–49, 52, 54, 56, 61, 62, 68, 70–72, 74, 76–78, 80, 83, 87, 89–94, 96, 97, 99, 105–113, 115, 118–121, 123, 125), 16 in central China (27, 31, 38, 50, 51, 53, 65, 67, 69, 85, 98, 100, 101, 117, 122, 126), and 31 in western China (24, 25, 28, 30, 33, 34, 41, 45, 55, 57–60, 63, 64, 66, 73, 75, 79, 81, 82, 84, 86, 88, 95, 102–104, 114, 116, 124).

The screening-positive analysis included 6,045,953 participants, of whom 226,212 screened positive. Sample sizes for individual effect sizes ranged from 640 to 1,793,787, and observed screening-positive rates ranged from 0.47% to 12.61%. The primary analysis of Cobb angle–confirmed prevalence included 1,162,720 participants and 11,147 confirmed cases. Sample sizes for individual studies ranged from 764 to 665,333, and observed prevalence ranged from 0.16% to 6.75%. Radiographic referral and completion were variably reported across the 23 studies. One study explicitly documented complete radiographic follow-up, five explicitly documented incomplete radiographic follow-up, and two provided partial pathway information that did not permit a strict radiographic completion rate. In the remaining 15 studies, radiographic completion could not be determined from the published report. Study-level information on radiographic referral and completion is provided in Supplementary Table S2. The included studies were based on school-based or general-population samples rather than populations selected for known secondary causes of scoliosis. However, etiological subtypes were not consistently reported in the primary studies; therefore, the pooled outcomes were retained as overall scoliosis, and separate pooled analyses of idiopathic and non-idiopathic scoliosis were not performed. With regard to etiological classification, 16 of the 103 included studies (15.5%) specifically targeted adolescent idiopathic scoliosis, idiopathic scoliosis, or potential AIS in their screening or outcome definitions; 85 studies (82.5%) reported scoliosis or spinal curvature abnormalities without clearly specifying an etiological subtype; and two studies (1.9%) explicitly differentiated idiopathic from non-idiopathic scoliosis. Accordingly, the pooled outcomes were retained as overall scoliosis rather than uniformly reclassified as AIS.

### 3.3 Methodological quality assessment

All 103 studies were evaluated using the JBI Critical Appraisal Checklist for Studies Reporting Prevalence Data. Quality assessment was performed once at the independent-study level for all 103 unique studies, irrespective of whether a study contributed screening-positive outcomes, radiographically confirmed outcomes, or both. Study participants and settings were adequately described in all studies (Q4: 103/103, 100.0%). Valid methods were used to identify the condition in 100 studies (Q6: 97.1%), and the condition was measured in a standard and reliable manner in 86 studies (Q7: 83.5%). The sampling frame was judged appropriate in 79 studies (Q1: 76.7%), and participants were sampled appropriately in 78 studies (Q2: 75.7%).

After reassessment using the predefined operational criteria, all 103 studies were rated Yes for Q3 (sample-size adequacy). The absence of a reported *a priori* sample-size calculation was not, by itself, considered grounds for an Unclear rating; judgments were based on the achieved sample size, study design and population coverage, and the adequacy of the sample for prevalence estimation. The main remaining methodological limitations concerned response-rate reporting and statistical analysis. For Q9, 64 studies (62.1%) were rated Unclear and eight (7.8%) were rated No, mainly because the eligible population, participation numbers, refusals, losses to follow-up, or response rates were incompletely reported. For Q8, 22 studies (21.4%) were rated No and one study (1.0%) was rated Unclear because of inconsistencies in case counts, prevalence estimates, or regression results, extrapolation from incomplete follow-up, or inadequate handling of non-response and loss to follow-up. Figure 2 presents the study-level judgments and the distribution of ratings across checklist items.

**Figure 2:**
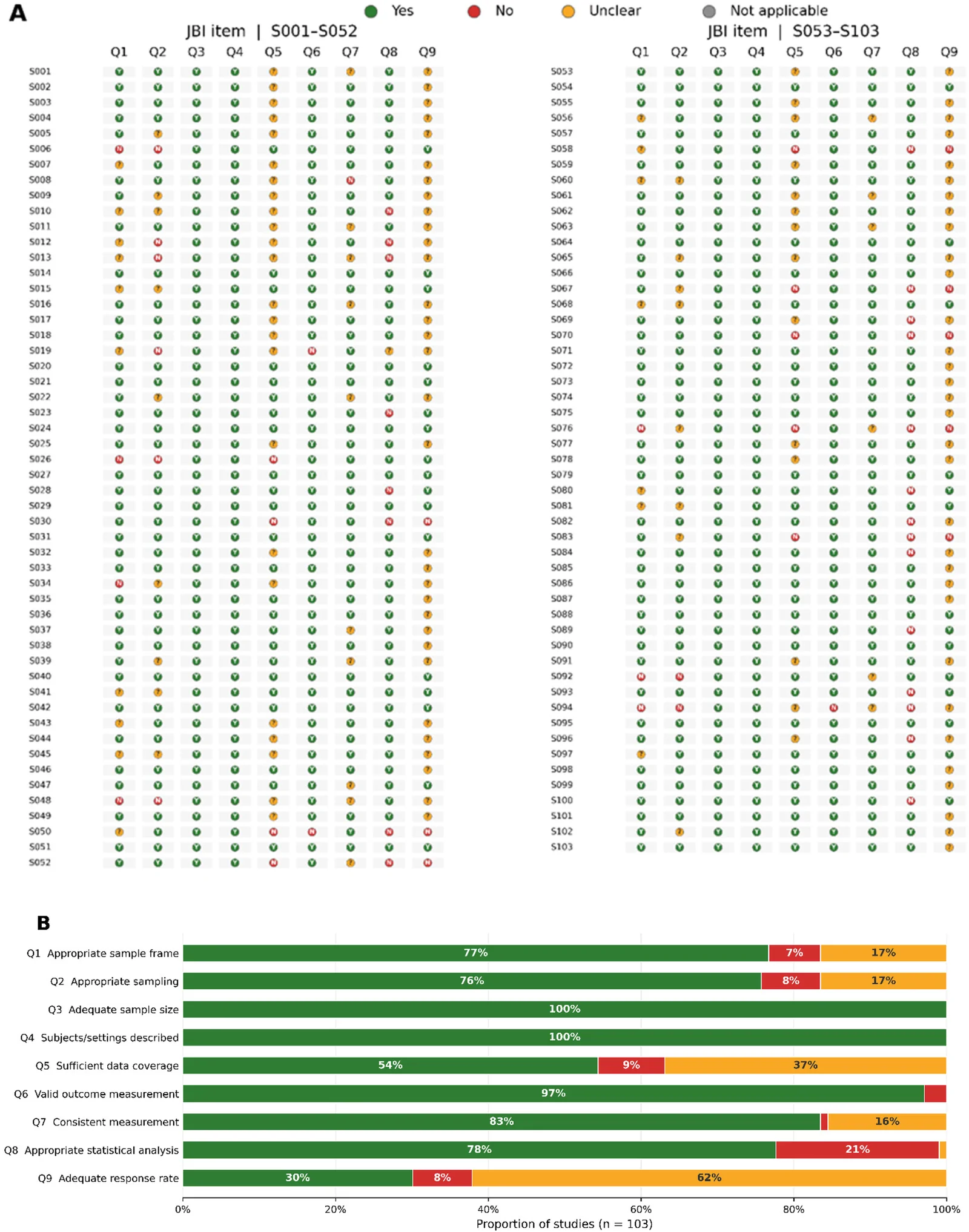
A methodological quality assessment of included studies: **(A)** JBI traffic light plot by study; **(B)** the proportion of each item assessment, *n* = 103.

### 3.4 Main analysis

Across 95 effect sizes, the pooled screening-positive rate was 3.43% (95% CI: 2.98%–3.93%; *I*^2^ = 99.84%; *τ*^2^ = 0.500; Q = 60,566.65; *P* < 0.001; 95% PI: 0.86%–12.70%). Across 23 studies, the pooled Cobb angle–confirmed prevalence was 1.83% (95% CI: 1.17%–2.86%; *I*^2^ = 99.85%; *τ*^2^ = 1.225; *Q* = 15,090.47; *P* < 0.001; 95% PI: 0.18%–16.41%) (Figures 3,4).

**Figure 3:**
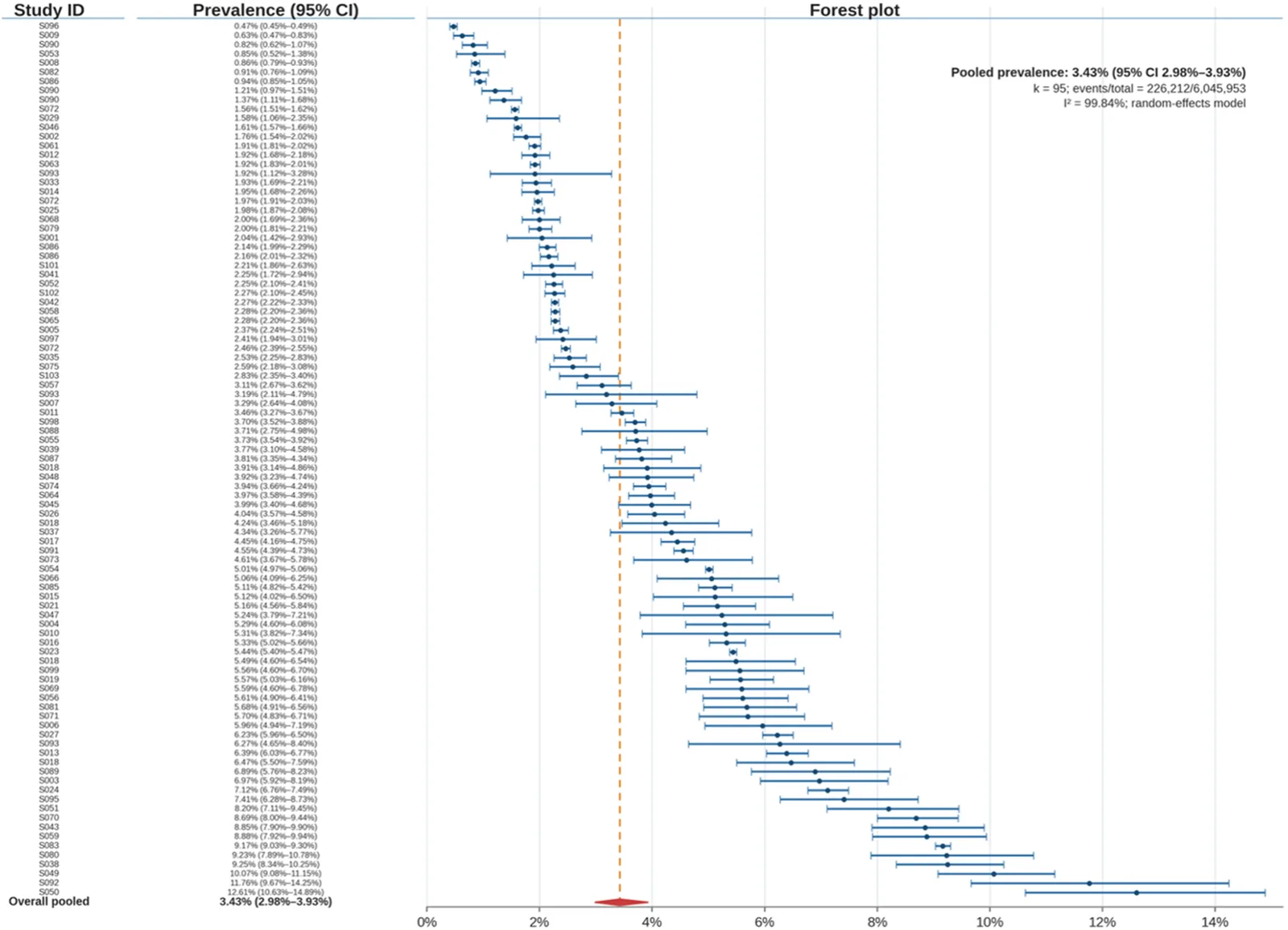

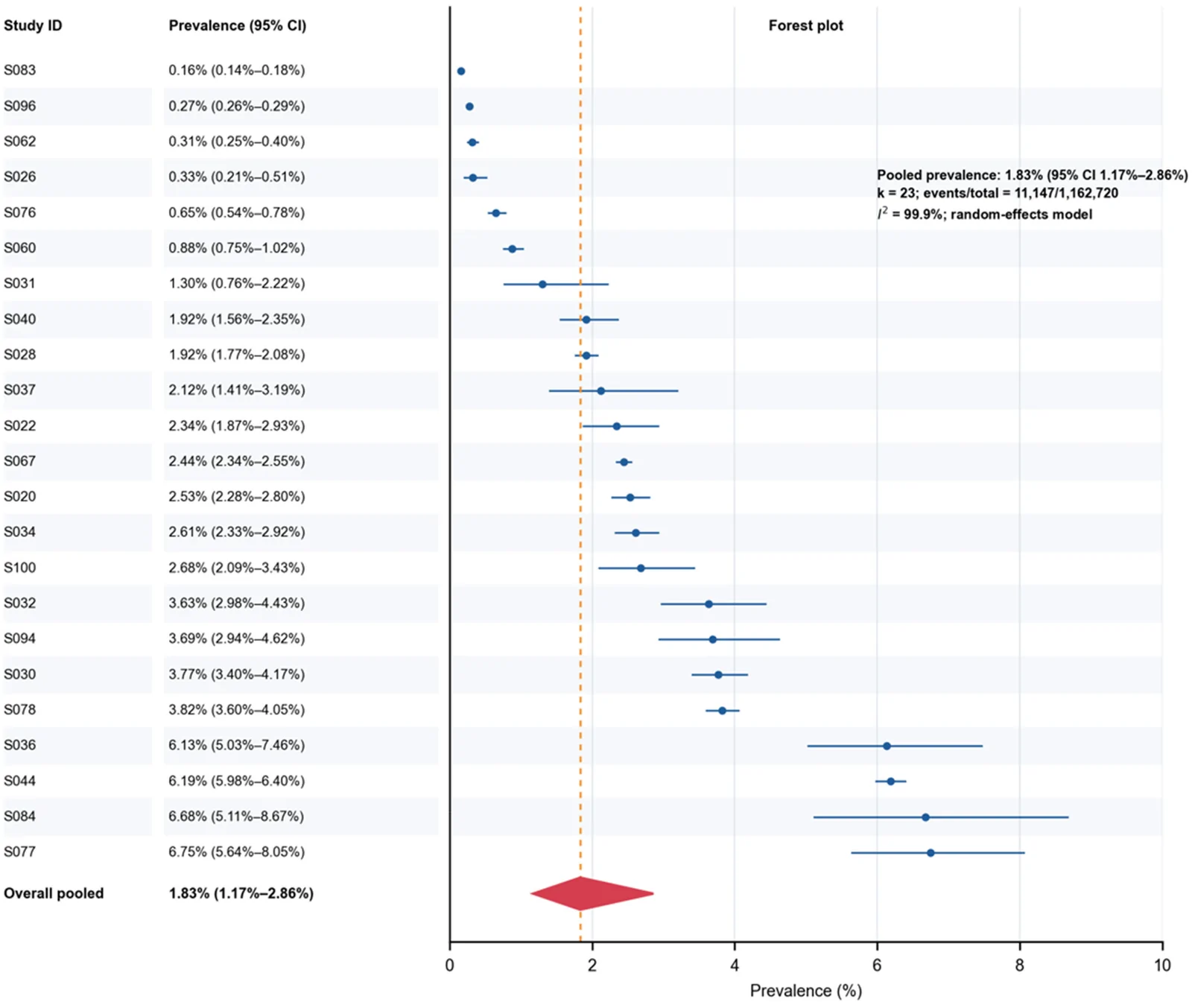
Forest plots of the primary analyses: **(A)** screening-positive rate, 95 effect sizes from 84 studies; **(B)** Cobb angle–confirmed prevalence, 23 studies. Study IDs (S001–S103) correspond to the master numbering of all 103 included studies and therefore are not sequential within each outcome-specific analysis.

**Figure 4:**
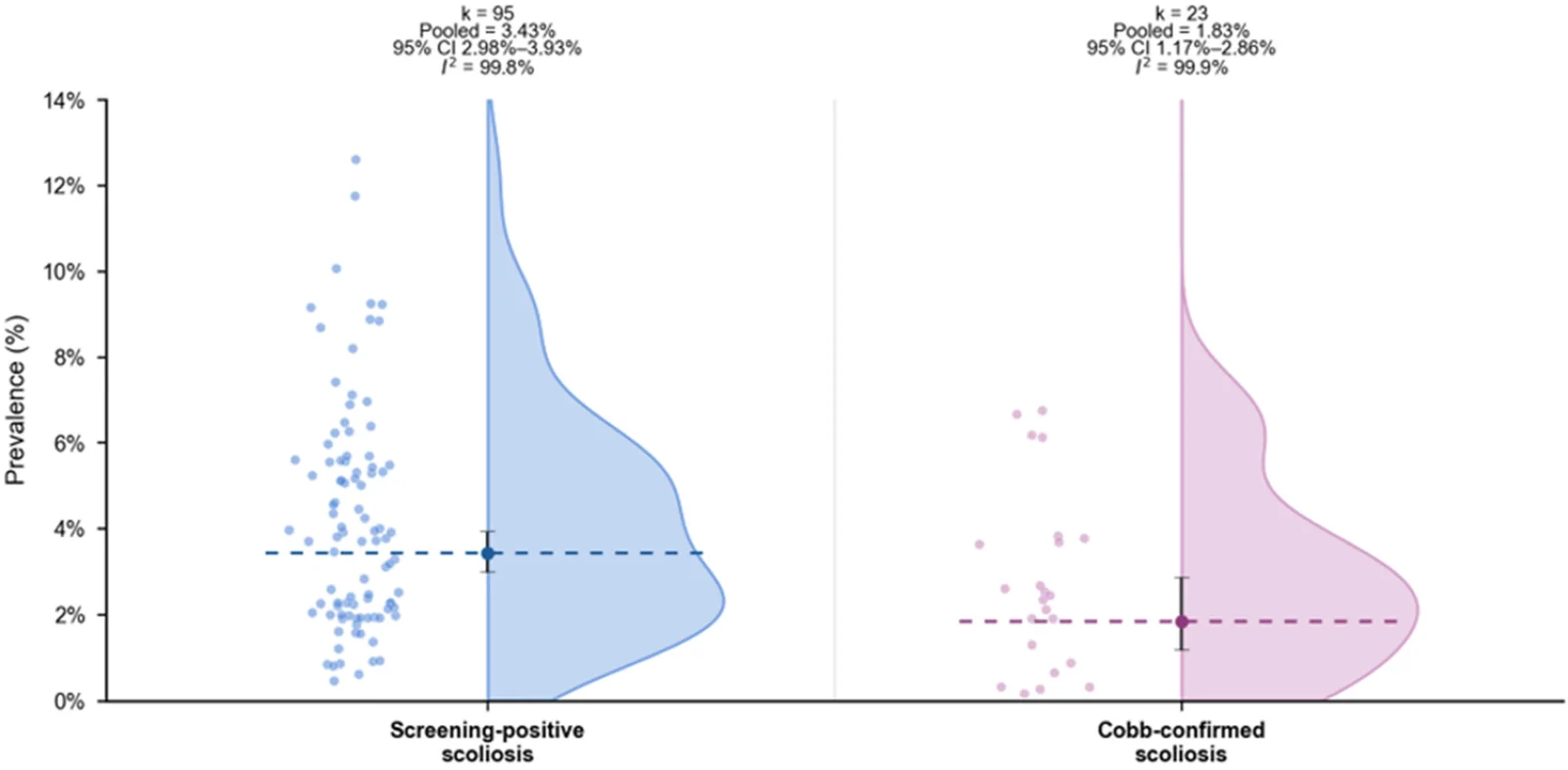
Distribution of study estimates and pooled results for screening positivity and Cobb angle–confirmed prevalence.

Because screening positivity and radiographically confirmed prevalence represent different diagnostic stages, they were not formally compared.

### 3.5 Analysis by screening framework and diagnostic stage

The pooled screening-positive rate was 2.14% (95% CI: 1.45%–3.16%) among studies classified as clinical/postural screening, 3.18% (95% CI: 2.34%–4.30%) among studies explicitly reporting screening according to GB/T 16133-2014, and 3.97% (95% CI: 3.41%–4.62%) among studies classified as ATR-based screening. The pooled Cobb angle–confirmed prevalence was 1.83% (95% CI: 1.17%–2.86%). Heterogeneity remained high within all groups. Because individual screening components were frequently used in combination and these subgroup estimates were derived from different studies rather than direct within-population comparisons, the observed differences should be interpreted as descriptive study-level variation and not as evidence of differences in diagnostic performance between screening approaches.

### 3.6 Subgroup analyses by sex, school stage, and urbanicity

Among 56 studies reporting sex-specific screening results, pooled rates were 2.85% (95% CI: 2.32%–3.49%) in boys and 4.60% (95% CI: 3.86%–5.47%) in girls; the within-study female-to-male prevalence ratio (PR) was 1.58 (95% CI: 1.41–1.77; *P* < 0.001).

Among 21 studies reporting sex-specific Cobb angle–confirmed prevalence, pooled estimates were 1.33% (95% CI: 0.80%–2.20%) in boys and 2.04% (95% CI: 1.28%–3.24%) in girls; the within-study PR was 1.49 (95% CI: 1.26–1.76; *P* < 0.001) (Figure 5A).

**Figure 5:**
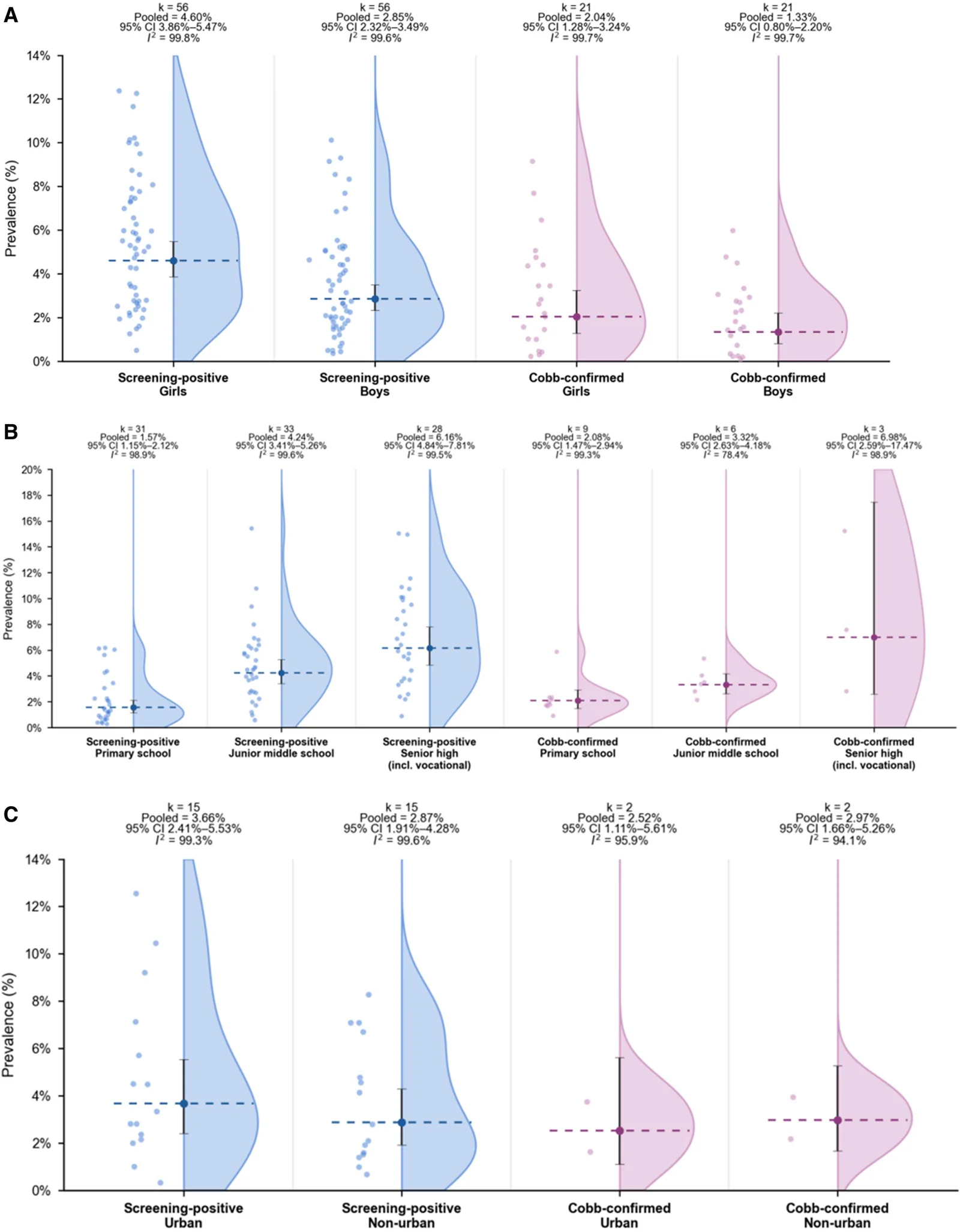
**(A)** Sex-stratified results for screening positivity and Cobb angle–confirmed prevalence. **(B)** School-stage-stratified results for primary, junior secondary, and senior secondary school. **(C)** Urban and non-urban subgroup results.

Screening-positive rates were 1.57% (95% CI: 1.15%–2.12%), 4.24% (95% CI: 3.41%–5.26%), and 6.16% (95% CI: 4.84%–7.81%) in primary, junior secondary, and senior secondary school, respectively. The corresponding Cobb angle–confirmed prevalence estimates were 2.08% (95% CI: 1.47%–2.94%), 3.32% (95% CI: 2.63%–4.18%), and 6.98% (95% CI: 2.59%–17.47%). Point estimates increased across school stages, although the groups were derived mainly from different studies (Figure 5B).

Among 15 studies with paired urban and non-urban screening data, pooled rates were 3.66% (95% CI: 2.41%–5.53%) and 2.87% (95% CI: 1.91%–4.28%), respectively; the within-study PR was 1.38 (95% CI: 1.06–1.80; *P* = 0.017). Only two studies reported paired confirmed data, with pooled estimates of 2.52% (95% CI: 1.11%–5.61%) in urban students and 2.97% (95% CI: 1.66%–5.26%) in non-urban students (Figure 5C).

### 3.7 Regional subgroup analysis

Pooled screening-positive rates were 3.57% (95% CI: 3.03%–4.21%) in eastern China, 3.64% (95% CI: 2.12%–6.19%) in central China, and 3.07% (95% CI: 2.23%–4.22%) in western China. Meta-regression did not detect regional differences (overall *P* = 0.566) (Figure 6).

**Figure 6:**
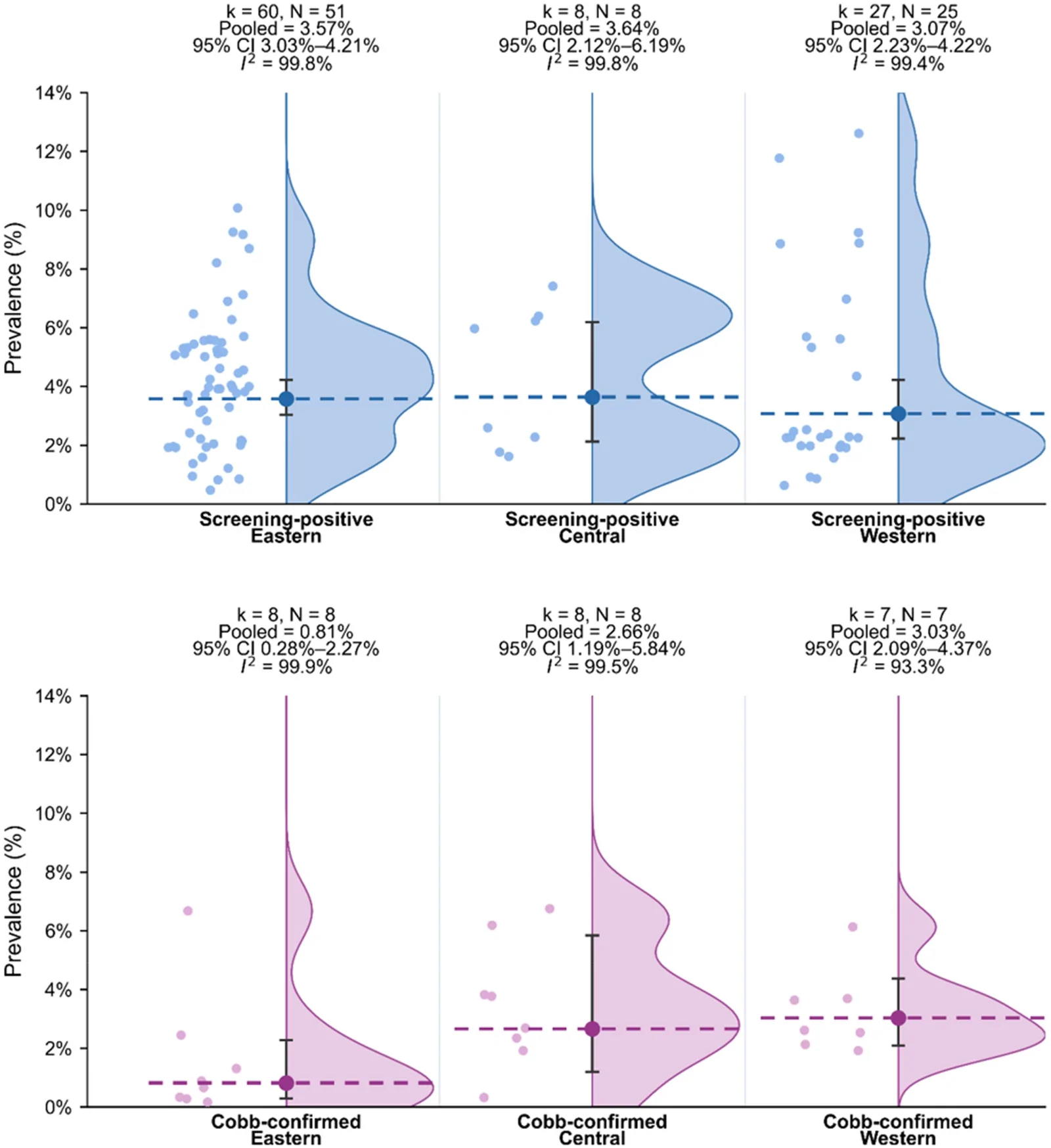
Regional subgroup results for eastern, central, and western China. k, number of effect sizes; N, number of independent studies.

Cobb angle–confirmed prevalence was 0.81% (95% CI: 0.28%–2.27%) in eastern China, 2.66% (95% CI: 1.19%–5.84%) in central China, and 3.03% (95% CI: 2.09%–4.37%) in western China. Region was associated with study-level prevalence (overall *P* = 0.011) and explained 24.5% of between-study variance. With eastern China as the reference, logit-prevalence coefficients were 1.212 for central China and 1.341 for western China. These ecological associations were not interpreted as causal.

### 3.8 Sensitivity analysis

Both outcomes remained stable across the sensitivity analyses (Figure 7).

**Figure 7:**
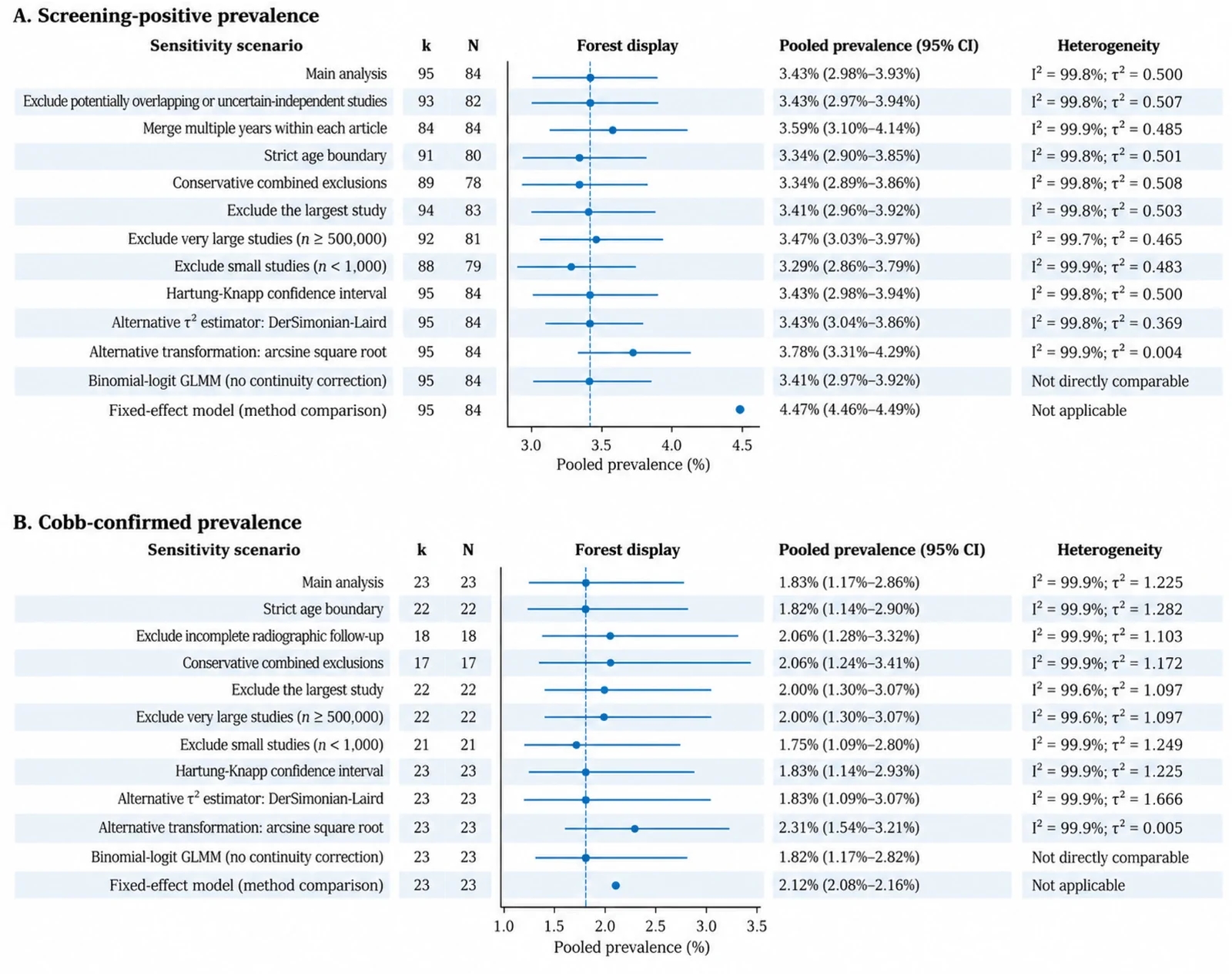
Main sensitivity analyses: **(A)** screening-positive rate; **(B)** Cobb angle–confirmed prevalence.

For screening positivity, leave-one-study-out estimates ranged from 3.37% to 3.55%. Estimates were similar after excluding potentially overlapping or non-independent studies (3.43%), combining multiple survey years within articles (3.59%), applying stricter age criteria (3.34%), excluding the largest study (3.41%), excluding studies with denominators ≥500,000 (3.47%), and excluding studies with sample sizes <1,000 (3.29%) (Supplementary Figure S1A).

In the additional analysis by screening program setting, 44 screening-positive studies were classified as routine/official public-health screening, 36 as investigator-initiated/local academic studies, and four as unclear. The pooled screening-positive rate was 3.35% (95% CI: 2.79%–4.02%) in routine/official screening programs and 3.82% (95% CI: 2.98%–4.89%) in investigator-initiated/local academic studies, with no evidence of a between-setting difference (*P* = 0.392). After excluding the four studies with unclear program setting, the pooled estimate across the remaining 80 studies was 3.55% (95% CI: 3.06%–4.13%), and substantial heterogeneity remained within both settings. Thus, the overall screening-positive estimate and the observed heterogeneity were not materially explained by screening program setting alone. Among the 23 studies reporting radiographically confirmed prevalence, only two were classified as routine/official public-health screening, compared with 20 investigator-initiated/local academic studies and one unclear study; therefore, a setting-stratified comparison for this outcome was considered too sparse for stable inference.

For Cobb angle–confirmed prevalence, leave-one-study-out estimates ranged from 1.73% to 2.05% (Supplementary Figure S1B). After excluding five studies with explicitly documented incomplete radiographic follow-up, 18 studies remained, comprising 854,613 participants and 7,952 confirmed cases. The pooled prevalence was 2.06% (95% CI: 1.28%–3.32%; *I*^2^ = 99.87%; *τ*^2^ = 1.103; 95% PI: 0.21%–17.21%), compared with 1.83% in the primary analysis. In the binomial-logit GLMM fitted directly to the study-level event counts and denominators without continuity correction, the pooled screening-positive rate was 3.41% (95% CI: 2.97%–3.92%), compared with 3.43% (95% CI: 2.98%–3.93%) in the primary analysis. The corresponding radiographically confirmed prevalence was 1.82% (95% CI: 1.17%–2.82%), compared with 1.83% (95% CI: 1.17%–2.86%) in the primary analysis. The GLMM estimates were therefore closely concordant with the primary estimates.

Hartung–Knapp adjustment did not change either point estimate; the screening-positive estimate was 3.43% (95% CI: 2.98%–3.94%) and the confirmed-prevalence estimate was 1.83% (95% CI: 1.14%–2.93%). For confirmed prevalence, estimates were 1.83% (95% CI: 1.09%–3.07%) using DerSimonian–Laird estimation, 2.31% (95% CI: 1.54%–3.21%) using the arcsine square-root transformation, and 2.12% (95% CI: 2.08%–2.16%) under a fixed-effect model. The fixed-effect model was used only for methodological comparison.

Overall, the main estimates were stable across alternative eligibility criteria, sample restrictions, transformations, and model specifications.

### 3.9 Meta regression

#### 3.9.1 Univariable meta-regression

For screening positivity, survey year was not associated with the outcome (*P* = 0.390; Supplementary Figure S2.1). Log sample size was negatively associated with screening positivity (*β* = −0.153, 95% CI: −0.229 to −0.076; *P* < 0.001) and explained 15.4% of between-study variance (Supplementary Figure S2.2). Region (overall *P* = 0.566; Supplementary Figure S2.3) and screening framework classification (overall *P* = 0.133; Supplementary Figure S2.4) were not associated with screening positivity.

For Cobb angle–confirmed prevalence, survey year was not associated with the outcome (*P* = 0.402; Supplementary Figure S3.1). Log sample size was negatively associated with prevalence (*β* = −0.346, 95% CI: −0.568 to −0.124; *P* = 0.002) and explained 28.1% of between-study variance (Supplementary Figure S3.2). Region was also associated with prevalence (overall *P* = 0.011), explaining 24.5% of between-study variance (Supplementary Figure S3.3).

#### 3.9.2 Multivariable meta-regression

The screening-positivity model included log sample size, screening framework classification, and region. The moderator test was significant (QM = 19.47, df = 5; *P* = 0.002), and the model explained 15.6% of between-study variance (residual *τ*^2^ = 0.409). Log sample size remained negatively associated with screening positivity (*β* = −0.146, 95% CI: −0.223 to −0.068; *P* < 0.001), whereas screening framework classification and region were not associated with the outcome.

The confirmed-prevalence model included log sample size and region. The moderator test was significant (QM = 17.00, df = 3; *P* < 0.001), and the model explained 39.4% of between-study variance (residual *τ*^2^ = 0.743). Log sample size remained negatively associated with prevalence (*β* = −0.274, 95% CI: −0.497 to −0.051; *P* = 0.016). Relative to eastern China, the coefficient was 1.034 for central China (95% CI: 0.169–1.899; *P* = 0.019) and 0.866 for western China (95% CI: −0.100 to 1.831; *P* = 0.079). Radiographic completion was not included as a meta-regression covariate because only a limited number of studies reported directly comparable completion data and the available measures referred to different stages of the referral pathway.

### 3.10 Three-level meta-analysis and effect size dependency

Five of the 84 independent studies contributing to the screening-positive analysis reported multiple survey years or analytical samples, resulting in 95 effect sizes in total. A three-level model nesting these 95 effect sizes within 84 independent studies yielded a pooled screening-positive rate of 3.56% (95% CI: 3.08%–4.12%; 95% PI: 0.92%–12.87%). The corresponding estimates were 3.43% in the conventional two-level model and 3.59% after combining multiple estimates within articles (Figure 8A). The three-level meta-analysis yielded level-specific *I*^2^ values of 24.17% for within-study heterogeneity and 75.71% for between-study heterogeneity, indicating that between-study variation was the predominant source of heterogeneity.

**Figure 8:**
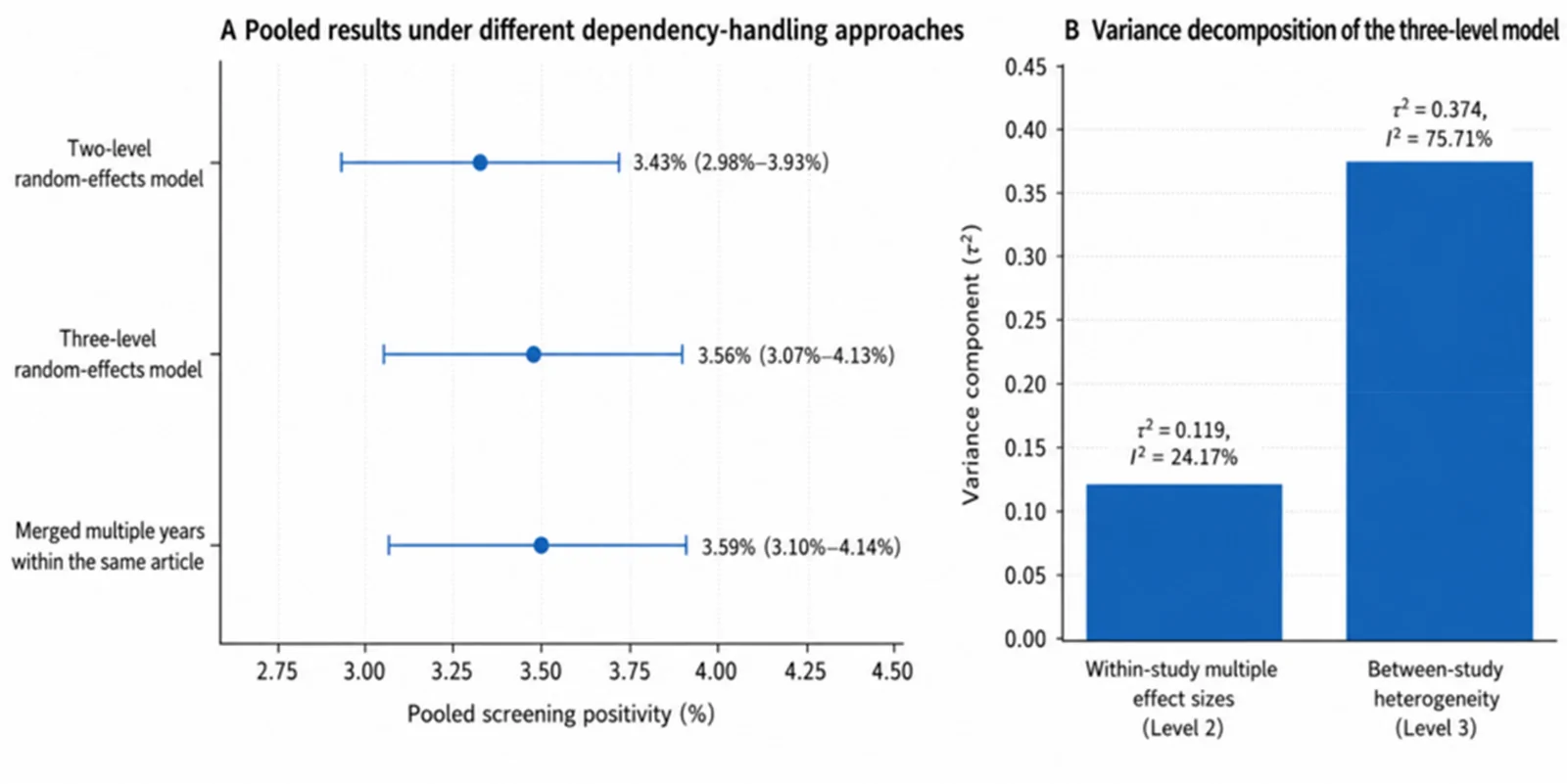
Three-level meta-analysis and variance decomposition for screening positivity based on 95 effect sizes nested within 84 independent studies: **(A)** two-level, three-level, and within-article merged estimates; **(B)** within-study and between-study variance components.

### 3.11 Influence diagnostics for the multivariable meta-regression models

The screening-positivity meta-regression and influence diagnostics were conducted at the independent-study level, with 84 study-level observations after aggregation of multiple effect sizes within the same study. In the screening-positivity model, no study exceeded the thresholds for Cook's distance or absolute DFBETAS. Study S042 had leverage slightly above the 3p/k threshold, and S009, S053, S082, S083, and S096 had absolute externally studentized residuals > 1.96. In leave-one-study-out analyses, the log sample-size coefficient ranged from −0.173 to −0.122 and remained significant in all 84 iterations (Figure 9A).

**Figure 9:**
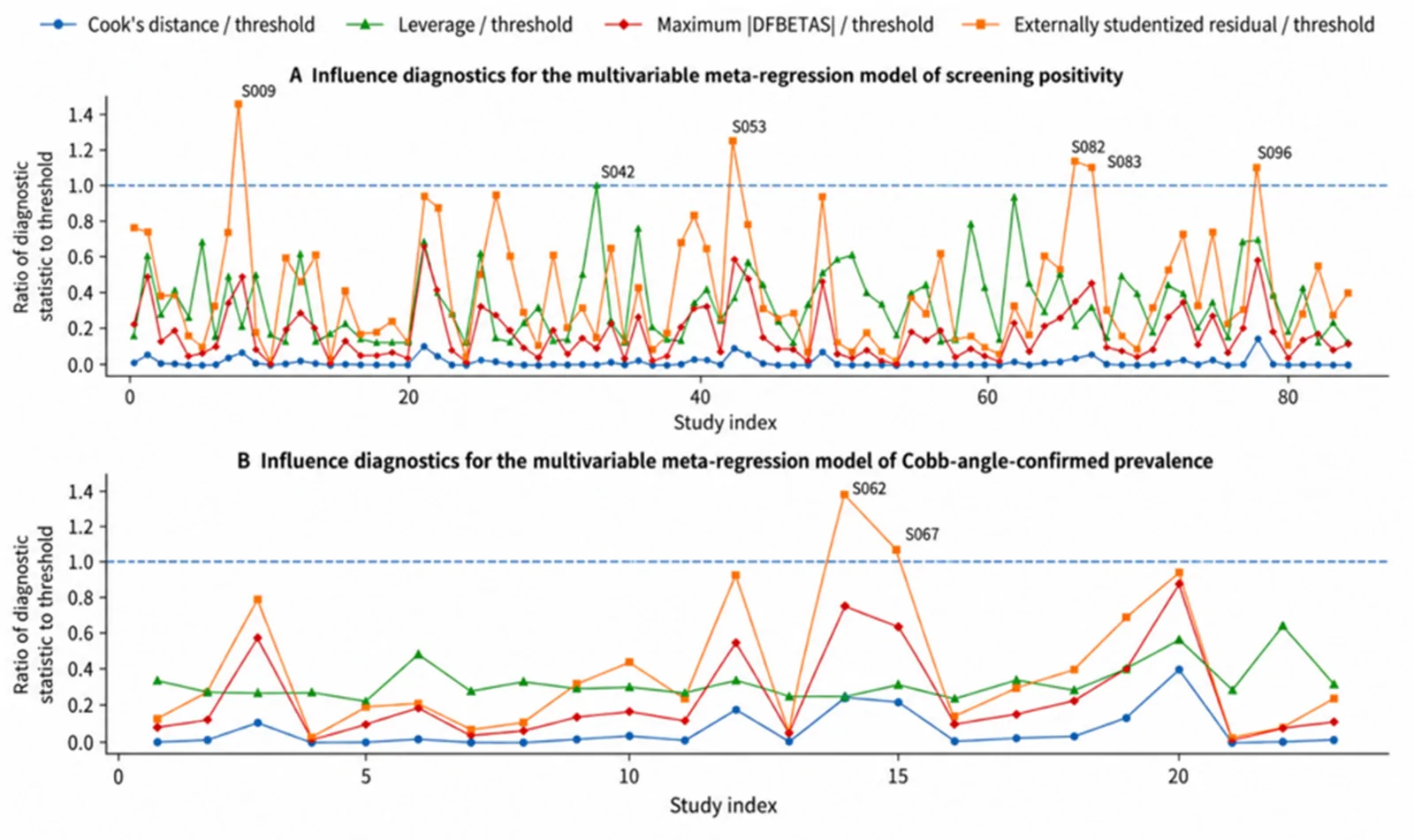
Influence diagnostics for the final multivariable meta-regression models: **(A)** screening-positivity model, 84 independent studies; **(B)** Cobb angle–confirmed model, 23 independent studies; dashed line, reference threshold for each standardized diagnostic measure. Study IDs correspond to the master numbering of the 103 included studies.

In the confirmed-prevalence model, no study exceeded the thresholds for Cook's distance, leverage, or DFBETAS. Studies S062 and S067 had absolute externally studentized residuals > 1.96. Coefficient directions remained unchanged in all leave-one-study-out analyses; significance was retained in 20 of 23 iterations for log sample size, 21 of 23 for central versus eastern China, and 3 of 23 for western versus eastern China (Figure 9B).

## 4 Discussion

### 4.1 Main findings

This review included 103 independent studies reporting recent scoliosis screening data for Chinese children and adolescents. Among surveys conducted during 2020–2025, the pooled screening-positive rate was 3.43%, whereas Cobb angle–confirmed prevalence was 1.83%. Within-study female-to-male PRs exceeded 1 for both outcomes. Point estimates increased from primary to junior secondary and senior secondary school; however, school-stage groups were drawn mainly from different studies, and therefore, this pattern does not establish an age-related increase. Studies using ATR measurement reported higher screening-positive rates than studies using postural observation or national-standard screening, but the methods were not compared in the same students and the difference cannot be attributed to the tool itself. Geographic region was associated with radiographically confirmed prevalence but not with initial screening positivity.

Both primary analyses showed substantial between-study heterogeneity (*I*^2^ = 99.84% for screening positivity and *I*^2^ = 99.85% for radiographically confirmed prevalence). The pooled screening-positive rate was 3.43% (95% PI: 0.86%–12.70%), and the radiographically confirmed prevalence was 1.83% (95% PI: 0.18%–16.41%). Confidence intervals and prediction intervals convey different information: confidence intervals quantify uncertainty around the pooled average prevalence, whereas prediction intervals indicate the range within which the true prevalence of a future comparable study may lie after accounting for between-study heterogeneity (11, 127). Therefore, the pooled estimates of 3.43% and 1.83% should be interpreted as average prevalence estimates across the populations represented by the included studies rather than as fixed national prevalence values. The wide prediction intervals may reflect differences in age and school-stage composition, screening criteria and thresholds, regional sampling, examination procedures, and completeness of radiographic follow-up. Although geographic region was associated with radiographically confirmed prevalence in the subgroup and meta-regression analyses, substantial residual heterogeneity remained, indicating that geographic differences alone did not account for the observed variability. Differences in screening implementation, diagnostic criteria, referral procedures, and completeness of radiographic follow-up may also have contributed to the remaining between-study variation. Clinically, these prediction intervals should not be interpreted as individual-level risk ranges or diagnostic thresholds; rather, they indicate that prevalence estimates in future comparable populations may differ substantially from the pooled averages.

The main estimates changed little under alternative inclusion rules, age limits, sample ranges, statistical treatments, and random-effects specifications. Thus, the estimates of 3.43% and 1.83% showed limited sensitivity to the analytical choices examined, although heterogeneity remained high.

### 4.2 Screening stages and confirmation pathways

The screening-positive rate and radiographically confirmed prevalence represent different stages of the scoliosis screening pathway. Postural examination, the Adams forward-bend test, GB/T 16133-2014, and angle of trunk rotation (ATR) measurements are primarily used to identify students with suspected scoliosis or those requiring further assessment, whereas standing spinal radiography and Cobb angle measurement are used for diagnostic confirmation (2–4). Accordingly, the screening-positive rate represents the proportion of students identified as positive during initial screening, whereas radiographically confirmed prevalence represents the proportion of confirmed cases among the total screened population. These outcomes have different epidemiological meanings and should not be interpreted as equivalent prevalence measures (2–5).

The pooled screening-positive rate of 3.43% in the present study was close to the 3.97% reported in a previous study of initial scoliosis screening in China. The radiographically confirmed prevalence of 1.83% was higher than the previous Chinese estimate of 1.20% and was close to the recent global estimate of 1.65% based on radiographic diagnosis (5, 6). Differences across studies may reflect variation in survey period, age composition, screening methods, diagnostic thresholds, case definitions, referral procedures, and completion of radiographic follow-up (2, 3, 5, 6, 128). A recent school-based study from Türkiye similarly showed that screening positivity and radiographic confirmation represent distinct stages of the scoliosis detection pathway (129).

In the subgroup analysis, studies classified as ATR-based screening showed higher pooled screening-positive rates than those classified under clinical/postural or national-standard screening. However, individual screening components such as postural examination, the Adams forward-bend test, and ATR measurement were frequently used in combination, particularly within standardized school-screening protocols. Therefore, these categories should be regarded as study-level analytical classifications rather than mutually exclusive clinical procedures. In addition, no direct within-population comparisons of the different screening approaches were available. Accordingly, the observed subgroup differences cannot be attributed to the screening procedures themselves or interpreted as evidence of differences in diagnostic performance (2–4). Radiographic outcomes should also distinguish among confirmed cases divided by the total screened population, confirmed cases divided by the number screening positive, and confirmed cases divided by the number undergoing radiography. Only the first denominator provides a population-level estimate of radiographically confirmed prevalence; the latter two primarily reflect referral and follow-up processes and should not be interpreted as population prevalence (2, 3, 5).

### 4.3 Gender and grade differences

In this study, both the screening-positive rate and radiographically confirmed prevalence were higher in girls than in boys, with female-to-male PRs of 1.58 and 1.49, respectively. Similar results have also been reported in recent school screening studies in different regions of China (24, 25). This difference may be related to gender differences in puberty growth and development, bone maturation, and the risk of curve progression (1, 128). However, the existing evidence mainly comes from observational studies, and therefore, it can only indicate an association between gender and the two outcomes but cannot infer a causal relationship.

The point estimates of both outcomes were higher in junior secondary and senior secondary school groups than in primary school groups. However, these estimates were derived mainly from cross-sectional data from different studies rather than longitudinal follow-up of the same individuals. Differences in age composition, school type, screening thresholds, and regional distribution may also have contributed to the observed pattern. Therefore, the higher prevalence estimates across school stages should be interpreted as cross-sectional differences between groups and do not demonstrate that scoliosis prevalence increases with age at the individual level.

### 4.4 Urban–rural and regional disparities

The within-study prevalence ratio for screening positivity was higher in urban than in non-urban populations. Previous Chinese studies on the differences between urban and rural areas did not yield consistent results, and the classification of urban, town, county, suburb, and rural areas often included different scenarios such as cities, towns, counties, suburbs, and urban–rural fringe (5, 130). In this study, “non-urban” also includes rural areas, suburbs, counties, and urban–rural fringe; therefore, the observed urban–non-urban difference may partly reflect differences in classification, population composition, and screening resources.

The screening-positive rates in eastern, central, and western China were broadly similar, whereas radiographically confirmed prevalence differed across regions. In the univariable analysis, region was associated with confirmed prevalence, and after adjustment for sample size, evidence of a difference remained for central versus eastern China, while the estimate for western China was less precise. However, these are study-level regional associations and should not be interpreted as evidence of differences in individual-level scoliosis risk. Regional categories may capture differences in population composition, economic and medical resources, screening coverage, access to radiography, referral procedures, and study design (5, 23, 130).

The importance of the referral pathway is illustrated by recent Chinese school-screening studies. In a 5-year screening program involving 90,635 adolescents in eastern China, 11.1% screened positive, but only 14.4% of screen-positive students underwent radiography; 559 cases were ultimately confirmed, corresponding to 0.62% of the total screened population. The authors specifically noted that incomplete radiographic follow-up could have resulted in underestimation of prevalence (131). Similarly, an earlier population-based school screening study in Shanghai reported that only 57.9% of screen-positive students completed hospital radiographic referral (132). In the present review, five of the 23 studies contributing to radiographically confirmed prevalence explicitly documented incomplete radiographic follow-up. After excluding these studies, the pooled confirmed prevalence increased from 1.83% (95% CI: 1.17%–2.86%) to 2.06% (95% CI: 1.28%–3.32%), while substantial heterogeneity remained (*I*^2^ = 99.87%; *τ*^2^ = 1.103). This modest change suggests that documented incomplete follow-up did not substantially alter the overall pooled estimate. However, radiographic completion could not be reliably determined in many of the remaining studies because referral denominators and actual radiography counts were incompletely or inconsistently reported. These findings suggest that part of the observed regional variation in radiographically confirmed prevalence may reflect differences in referral completion and access to imaging, rather than geographic differences in underlying scoliosis occurrence alone.

### 4.5 Heterogeneity, effect size dependence, and level-of-analysis explanations

The *I*^2^ values for both outcome measures in this study were close to 100%, indicating substantial differences in the screening-positive rates and radiographically confirmed prevalence estimates reported by different studies, and these differences could not be solely explained by sampling errors. Because of the generally large sample sizes and small intrastudy standard errors of the included studies, the *I*^2^ values could reach relatively high levels. Therefore, it is necessary to combine *τ*^2^ and prediction intervals for a comprehensive judgment (11–16). The pooled screening-positive rate was 3.43% (95% PI: 0.86%–12.70%), and the Cobb angle–confirmed prevalence was 1.83% (95% PI: 0.18%–16.41%). These prediction intervals were significantly wider than the corresponding confidence intervals, indicating that 3.43% and 1.83% represent only the average levels of the included studies and cannot be regarded as fixed values applicable to all regions, schools, and populations.

The three-level meta-analysis yielded level-specific *I*^2^ values of 24.17% for within-study heterogeneity and 75.71% for between-study heterogeneity, indicating that between-study variation was the predominant source of heterogeneity. The pooled screening-positive rate from the three-level model was 3.56%, which was close to the 3.43% from the conventional two-level model and the 3.59% obtained after aggregating multiple estimates within the same article, suggesting that effect-size dependence did not materially alter the overall conclusion (133, 134). Meta-regression showed an inverse association between sample size and both screening-positive and radiographically confirmed prevalence. This pattern should not be interpreted as evidence that sample size itself is related to scoliosis risk. Rather, study size may reflect differences in sampling frame, population coverage, study setting, screening implementation, and participant selection. To examine whether this association could be explained by differences between routine public-health screening programs and investigator-initiated studies, an additional sensitivity analysis was conducted according to screening program setting. The pooled screening-positive rate was 3.35% (95% CI: 2.79%–4.02%) among 44 routine/official public-health screening studies and 3.82% (95% CI: 2.98%–4.89%) among 36 investigator-initiated/local academic studies, with no evidence of a between-setting difference (*P* = 0.392). After excluding four studies with unclear program setting, the pooled estimate remained similar at 3.55% (95% CI: 3.06%–4.13%). These findings indicate that the inverse association between sample size and prevalence cannot be explained simply by differences between routine public-health screening programs and local academic surveys.

Considerable variation has also been reported among large-scale school-screening programs themselves. A recent population-based screening study of 69,811 students in Yunnan reported a screening-positive rate of 1.98%, whereas a 5-year program involving 90,635 adolescents in eastern China reported an initial screening-positive rate of 11.1% (24, 131). Previous systematic reviews of school scoliosis screening have likewise reported substantial heterogeneity across screening programs (135). These findings suggest that population composition, screening thresholds, examiner procedures, program implementation, and subsequent referral pathways may all contribute to between-study variation. Residual small-study and selection effects cannot be excluded, but sample size should not be interpreted as an individual-level risk factor or as the sole explanation for heterogeneity.

No Cook distance or DFBETAS exceeded the reference values, and the main regression coefficients direction did not change after individual studies were excluded. This indicates that the overall results were not dominated by a single or a few studies (136, 137). However, approximately 84.4% of the variance in screening-positive rates and approximately 60.6% of the variance in radiographically confirmed prevalence remained unexplained. The remaining heterogeneity might be related to factors such as age and puberty stage, gender composition, school type, screening threshold, examination personnel training, screening process, and X-ray review completion status (2, 3, 5, 6, 24). Therefore, the combined results of this study can be regarded as an overall average estimate but should be interpreted in combination with the prediction intervals and specific screening and diagnosis contexts.

### 4.6 Public health implications

The public health value of school screening depends on whether initial screening, re-examination, radiographic confirmation, and follow-up management form a continuous pathway. Relevant guidelines and evidence reviews indicate that screening itself is not the end point. Whether the positive screening results can receive appropriate re-examination, referral, diagnosis and subsequent management determines the actual value of the screening program (2, 3). Therefore, the monitoring report should clearly specify the diagnostic stage, denominator, screening tools and thresholds, referral coverage, actual number of X-ray examinations, and re-examination completion rate, in order to improve the comparability between regions and studies (2–5).

Girls and students in higher grades can be regarded as key populations to be focused on, but stratified management should still be combined with age, growth rate, bone maturity, previous screening results, family history, and clinical signs (1–3). The burden of diagnosed diseases and care not only involves the curve itself but may also affect the psychological and care experience of patients and their families (1, 138, 139). Therefore, the value of school screening should not only be measured by the positive rate but also include the completion rate of re-examination, the continuity of referral after diagnosis, stratified management, and family support.

### 4.7 Strengths and limitations

This study focuses on recent evidence based on the actual survey year, clearly distinguishes screening-positive outcomes from radiographically confirmed prevalence, verifies the denominators used for radiographic outcomes, systematically examines radiographic referral and follow-up information, and evaluates documented incomplete radiographic follow-up through a dedicated sensitivity analysis. In addition, prediction intervals, multiple sensitivity analyses, three-level meta-analysis, and multivariable influence diagnostics were used to assess the robustness of the findings.

These treatments respectively address the main methodological issues in the prevalence meta-analysis, such as high heterogeneity, reliance on effect sizes, and analysis of study-level moderating variables (11–18, 133, 134, 136). The results obtained from different dependency handling methods are consistent, which is the main advantage of this study.

This study still has limitations. First, the heterogeneity between studies for both outcome types is high, the prediction intervals are wide, and the existing meta-regression only explains part of the variance between studies. Therefore, the combined values can only represent the average level of the true rate distribution of the included studies and cannot represent the actual level in each region or school (13, 16). Second, whether there are repeated students in different years and the covariance between effect sizes are usually not reported. The three-level model can handle the clustering structure, but it cannot restore the unknown true covariance (133, 134). Third, radiographic referral and completion were inconsistently reported. Five of the 23 studies contributing to radiographically confirmed prevalence explicitly documented incomplete radiographic follow-up, whereas radiographic completion could not be reliably determined in many of the remaining studies because referral denominators or actual radiography counts were incompletely reported. Excluding the five studies with documented incomplete follow-up increased the pooled prevalence from 1.83% to 2.06%, but substantial heterogeneity remained. Because directly comparable radiographic completion percentages were available for too few studies, completion rate could not be reliably examined by meta-regression. Therefore, unreported or incomplete radiographic follow-up may still contribute to underestimation and residual between-study heterogeneity. Data on single-year age, ethnicity, school type, sampling procedures, screening thresholds, and examiner consistency were also incomplete, limiting further exploration of heterogeneity.

A sensitivity analysis excluding studies with “unclear” or “non-standard” screening thresholds was not performed because screening procedures were frequently combined across studies and no objective, consistently applicable criterion was available to classify these heterogeneous protocols as standard versus non-standard; introducing such a dichotomy would therefore have required an arbitrary *post hoc* classification.

Moreover, subgroup analyses by screening method relied mainly on comparisons across studies, rather than on direct within-population comparisons of different screening methods. Consequently, the observed differences in screening-positive rates may reflect differences in population characteristics, study settings, testing procedures, and positivity criteria. Such differences should not be interpreted as evidence of differences in the diagnostic performance of the various screening tools.

Radiographic confirmation was also not fully standardized across studies. Although all included radiographic outcomes were based on Cobb angle assessment, threshold definitions, radiographic acquisition protocols, and reporting of assessment procedures varied across studies. These differences may have affected the comparability of radiographically confirmed prevalence estimates across studies.

The meta-regression is based on study-level data and may still have residual confounding, collinearity, and ecological bias (17, 18); the Cobb angle model includes a small number of studies, and the stability of coefficients in some regions is limited. Although the review focused on general child and adolescent populations rather than populations selected for known secondary causes of scoliosis, etiological subtypes were not consistently reported across the primary studies. Only 16 of 103 studies (15.5%) specifically targeted AIS, idiopathic scoliosis, or potential AIS, whereas most studies reported non-differentiated scoliosis or spinal curvature abnormalities. Therefore, the pooled estimates should be interpreted as overall scoliosis estimates rather than specifically as the national prevalence of AIS.

Finally, although methodological quality was assessed using the JBI prevalence checklist, we did not perform a sensitivity analysis based on an overall quality category because the checklist does not provide a validated numerical score or threshold for defining lower-quality studies. Therefore, residual influence from methodological limitations across individual studies cannot be completely excluded.

## 5 Conclusion

Among Chinese children and adolescents surveyed during 2020–2025, the pooled screening-positive rate was 3.43%, and the radiographically confirmed prevalence rate was 1.83%. These outcomes represent different stages of the scoliosis screening pathway and should not be interpreted as equivalent measures. Both analyses showed extreme between-study heterogeneity, with wide 95% prediction intervals (0.86%–12.70% for screening positivity and 0.18%–16.41% for radiographically confirmed prevalence). Accordingly, the pooled estimates should be interpreted as random-effects averages across the heterogeneous populations represented by the included studies rather than as fixed national prevalence estimates applicable to all regions or schools in China. Although sample size and geographic region explained part of the between-study variation, substantial residual heterogeneity remained, and the regional meta-regression findings should be considered exploratory. Future studies should standardize terminology, denominators, screening thresholds, radiographic criteria, and reporting of referral and radiographic follow-up to improve comparability across studies.

## Data Availability

The raw data supporting the conclusions of this article will be made available by the authors, without undue reservation.
