# Supplementary material for: Screening positive rate and radiographically confirmed prevalence of scoliosis in Chinese children and adolescents from 2020 to 2025: a systematic review and meta-analysis
Source: Front Pediatr. 2026 Oct 30;14:1954010. doi: 10.3389/fped.2026.1954010 (PMC13759711; doi:10.3389/fped.2026.1954010)

Supplementary Materials

Supplementary Table S1. Complete Search Strategies for All Databases

Database	Field	Complete search formula
China National Knowledge Infrastructure (CNKI)	Topic	Subject = (Children OR Adolescents OR Primary and Secondary School Students OR Primary School Students OR Junior High School Students OR Senior High School Students OR Students) AND Subject = (Scoliosis OR Scoliosis Deformity OR Abnormal Spinal Curvature OR Deformed Spinal Curvature) AND Subject = (Screening OR Detection Rate OR Positive Rate OR Incidence Rate OR Epidemiology OR Adams Forward Bending Test OR Thoracic Rotation Angle OR ATR OR Cobb Angle OR X-ray OR Imaging)
Wanfang Data Knowledge Service Platform	Topic	Subject: (Children OR Adolescents OR Primary and Secondary School Students OR Elementary School Students OR Junior High School Students OR Senior High School Students OR Students) AND Subject: (Scoliosis OR Scoliosis Deformity OR Abnormal Spinal Curvature OR Deformed Spinal Curvature) AND Subject: (Screening OR Detection Rate OR Positive Rate OR Incidence Rate OR Epidemiology OR Adams Forward Bending Test OR Thoracic Rotation Angle OR ATR OR Cobb Angle OR X-ray OR Imaging)
PubMed	MeSH + Title/Abstract	(("Child"[Mesh] OR child*[tiab] OR adolescen*[tiab] OR student*[tiab] OR schoolchild*[tiab] OR "primary school"[tiab] OR "middle school"[tiab] OR "secondary school"[tiab]) AND ("Scoliosis"[Mesh] OR scoliosis[tiab] OR "spinal curvature"[tiab] OR "spinal curvature abnormal*"[tiab] OR "spinal deform*"[tiab]) AND ("Mass Screening"[Mesh] OR screen*[tiab] OR prevalence[tiab] OR epidemiolog*[tiab] OR "detection rate"[tiab] OR "Adams forward bend test"[tiab] OR "angle of trunk rotation"[tiab] OR ATR[tiab] OR radiograph*[tiab] OR "Cobb angle"[tiab]) AND ("China"[Mesh] OR China[tiab] OR Chinese[tiab]))
Embase（Elsevier）	Emtree + title/abstract/keyword	('child'/exp OR child*:ti,ab,kw OR adolescen*:ti,ab,kw OR student*:ti,ab,kw OR schoolchild*:ti,ab,kw) AND ('scoliosis'/exp OR scoliosis:ti,ab,kw OR 'spinal curvature':ti,ab,kw OR 'spinal curvature abnormality':ti,ab,kw OR 'spinal deformity':ti,ab,kw) AND ('mass screening'/exp OR 'prevalence'/exp OR screen*:ti,ab,kw OR prevalence:ti,ab,kw OR epidemiolog*:ti,ab,kw OR 'detection rate':ti,ab,kw OR 'adams forward bend test':ti,ab,kw OR 'angle of trunk rotation':ti,ab,kw OR atr:ti,ab,kw OR radiograph*:ti,ab,kw OR 'cobb angle':ti,ab,kw) AND ('china'/exp OR china:ti,ab,kw OR chinese:ti,ab,kw)
Scopus	TITLE-ABS-KEY	TITLE-ABS-KEY((child* OR adolescen* OR student* OR schoolchild* OR "primary school" OR "middle school" OR "secondary school") AND (scoliosis OR "spinal curvature" OR "spinal curvature abnormal*" OR "spinal deform*") AND (screen* OR prevalence OR epidemiolog* OR "detection rate" OR "Adams forward bend test" OR "angle of trunk rotation" OR ATR OR radiograph* OR "Cobb angle") AND (China OR Chinese))
Web of Science Core Collection	Topic (TS)	TS=((child* OR adolescen* OR student* OR schoolchild* OR "primary school" OR "middle school" OR "secondary school") AND (scoliosis OR "spinal curvature" OR "spinal curvature abnormal*" OR "spinal deform*") AND (screen* OR prevalence OR epidemiolog* OR "detection rate" OR "Adams forward bend test" OR "angle of trunk rotation" OR ATR OR radiograph* OR "Cobb angle") AND (China OR Chinese))

Note: All six databases were searched for studies published from January 1, 2020 to May 30, 2026, with publication language restricted to Chinese or English. The actual survey period (2020–2025) was verified during full-text screening. In addition, backward reference searching and forward citation searching were performed, and authors or experts were contacted where necessary to identify additional potentially eligible studies.

Supplementary Table S2A. Study characteristics and observed estimates (n = 103)

Screening-positive and radiographically confirmed outcomes are presented separately when both were reported within the same study. Study-specific observed estimates were calculated using the original numbers of cases and their corresponding denominators and do not represent the pooled estimates from the meta-analysis.

Study ID	Study	Survey year	Location	Study design	Population/age	Sample size	Screening/diagnostic method	Definition/threshold	Outcome	Cases/denominator	Observed estimate
S001 [26]	Zelong Miao et al. (2026)	2022	Haidian District, Beijing	School-based cross-sectional screening	Mixed primary, junior secondary, and senior secondary school students; 6–18 years	1,419	postural examination + Adams forward-bend test + angle of trunk rotation (ATR) measurement	ATR criterion defined in the original study	Screening-positive	29/1,419	2.04%
S002 [27]	Yanzhe Chen et al. (2026)	2024	Zhengzhou, Henan Province	School health surveillance (cross-sectional)	Mixed primary, junior secondary, and senior secondary school students; within the target age range of 6–18 years, inferred from school stage	11,526	postural examination + Adams forward-bend test + angle of trunk rotation (ATR) measurement + spinal mobility test	ATR criterion defined in the original study	Screening-positive	203/11,526	1.76%
S003 [28]	Jingwen Li et al. (2026)	2023	Guanghan, Deyang, Sichuan Province	School-based cross-sectional screening	Mixed primary, junior secondary, and senior secondary school students; generally within the target age range of 6–18 years, inferred from the primary/secondary school sample	1,951	postural examination + Adams forward-bend test + angle of trunk rotation (ATR) measurement + spinal mobility test	ATR criterion defined in the original study	Screening-positive	136/1,951	6.97%
S004 [29]	Chengcheng Xu et al. (2026)	2023	Yuhong District, Shenyang, Liaoning Province	School-based cross-sectional screening	Primary school students; within the target age range of 6–18 years	3,516	postural examination	Positive postural examination	Screening-positive	186/3,516	5.29%
S005 [30]	Hou Dong et al. (2026)	2024–2025	Gannan Tibetan Autonomous Prefecture, Gansu Province	School-based cross-sectional screening	Mixed primary, junior secondary, and senior secondary school students; within the target age range of 6–18 years	51,025	postural examination + Adams forward-bend test + angle of trunk rotation (ATR) measurement + electronic device-assisted assessment	ATR ≥5°	Screening-positive	1,211/51,025	2.37%

S006 [31]	Weiwei Xia et al. (2026)	2024	Huaibin County, Xinyang, Henan Province	School-based cross-sectional screening	Junior secondary school students; within the target age range of 6–18 years	1,710	Adams forward-bend test + angle of trunk rotation (ATR) measurement + electronic device-assisted assessment	ATR ≥5°	Screening-positive	102/1,710	5.96%
S007 [32]	Hui Feng et al. (2026)	2023	Dezhou, Shandong Province	School-based cross-sectional screening	Primary, junior secondary, and senior secondary school students; within the target age range of 6–18 years	2,374	postural examination + Adams forward-bend test + angle of trunk rotation (ATR) measurement	ATR ≥5°	Screening-positive	78/2,374	3.29%
S008 [33]	Tingting Li et al. (2026)	2024	Chanba Ecological District, Xi'an, Shaanxi Province	School-based cross-sectional screening	Primary and junior secondary school students; primary and junior secondary school students	64,981	postural examination + Adams forward-bend test	Positive postural examination	Screening-positive	558/64,981	0.86%
S009 [34]	Yong Zhao et al. (2026)	2022	Baoshan, Yunnan Province	School health surveillance (cross-sectional)	Primary, junior secondary, and senior secondary school students; within the target age range of 6–18 years; inferred from school stage	7,816	postural examination + Adams forward-bend test + angle of trunk rotation (ATR) measurement + spinal mobility test + prone-position test	ATR criterion defined in the original study	Screening-positive	49/7,816	0.63%
S010 [35]	Hao Jiang et al. (2022)	2020–2021	Lanshan District, Linyi, Shandong Province	School-based cross-sectional screening	Mixed primary and secondary school students; within the target age range of 6–18 years	640	postural examination + Adams forward-bend test + angle of trunk rotation (ATR) measurement + radiographic Cobb-angle measurement	Screening-positive criterion defined in the original study	Screening-positive	34/640	5.31%
S011 [36]	Fang Gu et al. (2024)	2021	Zhejiang Province	School-based cross-sectional screening	Mixed primary and secondary school students; within the target age range of 6–18 years	31,308	postural examination + Adams forward-bend test + angle of trunk rotation (ATR) measurement + spinal mobility test	ATR ≥5°	Screening-positive	1,084/31,308	3.46%
S012 [37]	Quan Liu et al. (2024)	2020–2023	Haicang District, Xiamen, Fujian Province	School-based cross-sectional screening	Mixed primary and secondary school students; 10–18 years	11,735	postural examination + Adams forward-bend test + angle of trunk rotation (ATR) measurement	ATR ≥5°	Screening-positive	225/11,735	1.92%
S013 [38]	Yixin Wen et al. (2025)	2023–2024	Hanyang District, Wuhan, Hubei Province	School-based cross-sectional screening	Mixed primary and secondary school students; age range not fully reported	16,898	postural examination + Adams forward-bend test + angle of trunk rotation (ATR) measurement	ATR >5°	Screening-positive	1,080/16,898	6.39%
S014 [39]	Jianru Cai et al. (2024)	2023	Nantong, Jiangsu Province	School-based cross-sectional screening	Mixed primary, secondary, and vocational high school students; 7–18 years	8,872	postural examination + Adams forward-bend test + angle of trunk rotation (ATR) measurement	ATR ≥5°	Screening-positive	173/8,872	1.95%
S015 [40]	Hongxin Chen et al. (2025)	2023–2024	Guangzhou, Guangdong Province	School-based cross-sectional screening	Mixed primary and junior secondary school students; within the target age range of 6–18 years	1,231	postural examination + Adams forward-bend test + angle of trunk rotation (ATR) measurement	ATR ≥5°	Screening-positive	63/1,231	5.12%
S016 [41]	Li Zhang et al. (2023)	2022	Nanhua County, Chuxiong Yi Autonomous Prefecture, Yunnan Province	School-based cross-sectional screening	Mixed primary, junior secondary, and senior secondary school students; within the target age range of 6–18 years	18,980	postural examination + Adams forward-bend test + angle of trunk rotation (ATR) measurement	ATR ≥5°	Screening-positive	1,011/18,980	5.33%
S017 [42]	Xiaoxiao Zhou et al. (2023)	2021	Taizhou, Zhejiang Province	School-based cross-sectional screening	Mixed primary, junior secondary, and senior secondary school students; within the target age range of 6–18 years	18,219	postural examination + Adams forward-bend test + angle of trunk rotation (ATR) measurement + spinal mobility test + prone-position test	ATR >5°	Screening-positive	810/18,219	4.45%
S018 [43]	Linyao Chen et al. (2024)	2020, 2021, 2022, 2023	Chenghai District, Shantou, Guangdong Province	School health surveillance (cross-sectional)	Mixed primary, junior secondary, and senior secondary school students; within the target age range of 6–18 years; inferred from grades 1–12	8,372	postural examination + Adams forward-bend test + angle of trunk rotation (ATR) measurement + spinal mobility test	ATR >5°	Screening-positive	423/8,372	5.05%
S019 [44]	Jun Jian et al. (2024)	2023	Zhoucun District, Zibo, Shandong Province	School-based cross-sectional screening	Mixed primary, junior secondary, and senior secondary school students; primary, junior secondary, and senior secondary school students	6,395	postural examination + Adams forward-bend test + angle of trunk rotation (ATR) measurement + spinal mobility test	ATR ≥5°	Screening-positive	356/6,395	5.57%
S020 [45]	Yunfeng Qiu et al. (2024)	2022–2023	Guiyang, Guizhou Province	School-based cross-sectional screening	Mixed primary and junior secondary school students; 10–15 years	14,005	postural examination + Adams forward-bend test + angle of trunk rotation (ATR) measurement + radiographic Cobb-angle measurement	Cobb angle ≥10°	Radiographically confirmed	354/14,005	2.53%
S021 [46]	Weidong Wang et al. (2024)	2024	An unspecified district of Taizhou, Zhejiang Province	School-based cross-sectional screening	Mixed primary and junior secondary school students; within the target age range of 6–18 years; inferred from grade level	4,593	postural examination + Adams forward-bend test + angle of trunk rotation (ATR) measurement + spinal mobility test	ATR ≥5°	Screening-positive	237/4,593	5.16%
S022 [126]	Zhanjun Zhang et al. (2025)	2022–2024	Yuzhou, Henan Province	School-based cross-sectional screening	Primary school students; within the target age range of 6–18 years	3,200	radiographic Cobb-angle measurement	Cobb angle >10°	Radiographically confirmed	75/3,200	2.34%
S023 [47]	Yujie Mei et al. (2025)	2023	Shenzhen, Guangdong Province	School-based cross-sectional screening	Mixed primary, junior secondary, and senior secondary school students; 6–19 years	1,793,787	postural examination + Adams forward-bend test + angle of trunk rotation (ATR) measurement + spinal mobility test	ATR ≥5°	Screening-positive	97,529/1,793,787	5.44%
S024 [48]	Zhongrui Peng et al. (2025)	2020	Xiamen, Fujian Province	School-based cross-sectional screening	Mixed primary, junior secondary, and senior secondary school students; within the target age range of 6–18 years	19,315	postural examination + Adams forward-bend test + angle of trunk rotation (ATR) measurement	Screening-positive criterion defined in the original study	Screening-positive	1,375/19,315	7.12%

S025 [24]	Shanshan Song et al. (2025)	2021	Yunnan Province	School-based cross-sectional screening	Mixed primary, junior secondary, and senior secondary school students; within the target age range of 6–18 years	69,811	postural examination + Adams forward-bend test + angle of trunk rotation (ATR) measurement + spinal mobility test	ATR ≥5°	Screening-positive	1,379/69,811	1.98%
S026 [49]	Xiao Chen et al. (2025)	2022	Shantou, Guangdong Province	School-based cross-sectional screening	Junior secondary school students; 12–16 years	5,814	Screening: postural examination + Adams forward-bend test + angle of trunk rotation (ATR) measurement Confirmation: postural examination + Adams forward-bend test + angle of trunk rotation (ATR) measurement + radiographic Cobb-angle measurement	Screening: ATR ≥5° Confirmation: Cobb angle ≥10°	Screening-positive; radiographically confirmed	Screening: 235/5,814 Confirmed: 19/5,814	Screening: 4.04% Confirmed: 0.33%
S027 [50]	Yanliang Jiao et al. (2025)	2024	Baohe District, Hefei, Anhui Province	School-based cross-sectional screening	Mixed primary, junior secondary, and senior secondary school students; within the target age range of 6–18 years, inferred from school stage	30,470	postural examination + Adams forward-bend test + angle of trunk rotation (ATR) measurement	ATR >7°	Screening-positive	1,897/30,470	6.23%
S028 [51]	Suping Jiang et al. (2025)	2023	Jiujiang, Jiangxi Province	School-based cross-sectional screening	Mixed primary, junior secondary, and senior secondary school students; within the target age range of 6–18 years, inferred from school stage	30,022	postural examination + Adams forward-bend test + radiographic Cobb-angle measurement	Cobb angle ≥10°	Radiographically confirmed	575/30,022	1.92%
S029 [52]	Yixian Li et al. (2025)	2024	An urban district of Shijiazhuang, Hebei Province	School-based cross-sectional screening	Mixed primary, junior secondary, and senior secondary school students; within the target age range of 6–18 years	1,516	postural examination + Adams forward-bend test + angle of trunk rotation (ATR) measurement + spinal mobility test	ATR ≥5°	Screening-positive	24/1,516	1.58%
S030 [53]	Kun Liu et al. (2025)	2023	Hanyang District, Wuhan, Hubei Province	School-based cross-sectional screening	Junior secondary school students; within the target age range of 6–18 years	9,501	postural examination + Adams forward-bend test + angle of trunk rotation (ATR) measurement + radiographic Cobb-angle measurement	Cobb angle ≥10° (incomplete radiographic follow-up)	Radiographically confirmed; included in primary meta-analysis and excluded in sensitivity analysis because radiographic follow-up was incomplete	358/9,501	3.77%
S031 [54]	Meilan Liu et al. (2025)	2025	Huaiyin District, Jinan, Shandong Province	School-based cross-sectional screening	Mixed primary and junior secondary school students; within the target age range of 6–18 years; inferred from school stage	998	postural examination + Adams forward-bend test + spinal mobility test + radiographic Cobb-angle measurement	Cobb angle ≥10°	Radiographically confirmed	13/998	1.30%
S032 [55]	Min Liu et al. (2025)	2023	Deyang, Sichuan Province	School-based cross-sectional screening	Primary school students; within the target age range of 6–18 years	2,559	postural examination + Adams forward-bend test + angle of trunk rotation (ATR) measurement + prone-position test + radiographic Cobb-angle measurement + electronic device-assisted assessment	Cobb angle ≥10°	Radiographically confirmed	93/2,559	3.63%
S033 [56]	Wenjuan Qi et al. (2025)	2022	Shanghai	School-based cross-sectional screening	Junior secondary school students; within the target age range of 6–18 years; inferred from school stage	10,710	postural examination + Adams forward-bend test + angle of trunk rotation (ATR) measurement + spinal mobility test	ATR ≥5°	Screening-positive	207/10,710	1.93%
S034 [57]	Tianlin Song et al. (2025)	2020–2021	Pastoral areas of Golog, Yushu, and Huangnan prefectures, Qinghai Province	School-based cross-sectional screening	Mixed kindergarten, primary, and secondary school students; 3–17 years; school-based subgroup aged 7–17 years extracted	11,078	postural examination + Adams forward-bend test + angle of trunk rotation (ATR) measurement + radiographic Cobb-angle measurement	Cobb angle ≥10°	Radiographically confirmed	289/11,078	2.61%
S035 [58]	Qin Su et al. (2025)	2024	Luzhou, Sichuan Province	School-based cross-sectional screening	Mixed primary, junior secondary, senior secondary, and vocational high school students; within the target age range of 6–18 years; inferred from school stage	11,326	postural examination + Adams forward-bend test + angle of trunk rotation (ATR) measurement + spinal mobility test + prone-position test	ATR ≥5°	Screening-positive	286/11,326	2.53%
S036 [59]	Fang Wang et al. (2025)	2024	Jiayuguan, Gansu Province	School-based cross-sectional screening	Junior and senior secondary school students; 10–18 years	1,500	postural examination + angle of trunk rotation (ATR) measurement + radiographic Cobb-angle measurement	Cobb angle ≥10°	Radiographically confirmed	92/1,500	6.13%
S037 [60]	Haonan Wang et al. (2025)	2022	Gongshan Derung and Nu Autonomous County, Yunnan Province	School-based cross-sectional screening	Primary and secondary school students; 5–18 years	1,036	Screening: postural examination + Adams forward-bend test + angle of trunk rotation (ATR) measurement + radiographic Cobb-angle measurement Confirmation: postural examination + Adams forward-bend test + angle of trunk rotation (ATR) measurement + radiographic Cobb-angle measurement	Screening: ATR ≥5° Confirmation: Cobb angle ≥10°	Screening-positive; radiographically confirmed	Screening: 45/1,036 Confirmed: 22/1,036	Screening: 4.34% Confirmed: 2.12%

S038 [61]	Yuanyuan Wang et al. (2025)	2022	Shanghai	School-based cross-sectional screening	Mixed primary, junior secondary, senior secondary, and vocational high school students; within the target age range of 6–18 years	3,546	postural examination + Adams forward-bend test + angle of trunk rotation (ATR) measurement + electronic device-assisted assessment	ATR ≥5°	Screening-positive	328/3,546	9.25%
S039 [62]	Yingying Wei et al. (2025)	2023	Chongchuan District, Nantong, Jiangsu Province	School health surveillance (cross-sectional)	Mixed primary, junior secondary, senior secondary, and higher vocational students; within the target age range of 6–18 years; inferred from school stage	2,575	postural examination + Adams forward-bend test + angle of trunk rotation (ATR) measurement + spinal mobility test	ATR >5°	Screening-positive	97/2,575	3.77%
S040 [63]	Rong Wei et al. (2025)	2021–2022	Jinfeng District, Shapotou District, Yanchi County, and Pingluo County, Ningxia Hui Autonomous Region	School-based cross-sectional screening	Primary and junior secondary school students; within the target age range of 6–18 years	4,595	postural examination + Adams forward-bend test + spinal mobility test + radiographic Cobb-angle measurement	Cobb angle ≥10°	Radiographically confirmed	88/4,595	1.92%
S041 [64]	Tao Li et al. (2025)	2023–2024	Xinshi District, Urumqi, Xinjiang Uyghur Autonomous Region	School-based cross-sectional screening	Junior secondary school students; 12–16 years	2,315	postural examination + Adams forward-bend test + angle of trunk rotation (ATR) measurement	Screening-positive criterion defined in the original study	Screening-positive	52/2,315	2.25%
S042 [65]	Fengming Xu et al. (2025)	2024	Henan Province	School-based cross-sectional screening	Mixed primary, junior secondary, and senior secondary school students; within the target age range of 6–18 years, inferred from school stage	255,765	postural examination + Adams forward-bend test + angle of trunk rotation (ATR) measurement + spinal mobility test + prone-position test	Screening-positive criterion defined in the original study	Screening-positive	5,812/255,765	2.27%
S043 [66]	Chen Zhang et al. (2025)	2023	Baiyin, Jinchang, Lanzhou, Linxia Hui Autonomous Prefecture, and Gannan Tibetan Autonomous Prefecture, Gansu Province	School-based cross-sectional screening	Mixed junior and senior secondary school students; 12–18 years	3,118	Adams forward-bend test + angle of trunk rotation (ATR) measurement	ATR >5°	Screening-positive	276/3,118	8.85%
S044 [67]	Le Zhao et al. (2025)	2021–2023	Urban area of Shiyan, Hubei Province	School-based cross-sectional screening	Mixed primary and junior secondary school students; within the target age range of 6–18 years, inferred from school stage	52,762	postural examination + Adams forward-bend test + spinal mobility test + prone-position test + radiographic Cobb-angle measurement	Cobb angle >10°	Radiographically confirmed	3,264/52,762	6.19%
S045 [68]	Weiliang Zheng et al. (2025)	2025	Minhou County, Fuzhou, Fujian Province	School-based cross-sectional screening	Mixed primary and junior secondary school students; within the target age range of 6–18 years, inferred from school stage	3,656	Adams forward-bend test + angle of trunk rotation (ATR) measurement + electronic device-assisted assessment	ATR ≥7°	Screening-positive	146/3,656	3.99%
S046 [69]	Yang Zhou et al. (2025)	2023	14 prefecture-level cities/prefectures in Hunan Province	School-based cross-sectional screening	Mixed primary, junior secondary, and senior secondary school students; within the target age range of 6–18 years	281,401	postural examination + Adams forward-bend test + angle of trunk rotation (ATR) measurement + spinal mobility test	ATR ≥5°	Screening-positive	4,540/281,401	1.61%
S047 [70]	Yujuan Chen et al. (2021)	2020	Shijiazhuang, Hebei Province	School-based cross-sectional screening	Kindergarten and primary school children; only the 6–8-year subgroup was extracted; the full sample included children aged 4–5 years	668	postural examination + Adams forward-bend test	ATR ≥5°	Screening-positive	35/668	5.24%
S048 [71]	Gang Li et al. (2021)	2021	Huizhou, Guangdong Province	School-based cross-sectional screening	Primary and junior secondary school students; within the target age range of 6–18 years; upper age limit above 14 years not reported	2,579	postural examination + Adams forward-bend test	ATR ≥5°	Screening-positive	101/2,579	3.92%
S049 [72]	Yang Liu et al. (2021)	2020	Longkou, Shandong Province	School-based cross-sectional screening	Junior secondary school students; secondary-school sample included 19-year-olds; included in sensitivity analysis	3,248	postural examination + Adams forward-bend test + angle of trunk rotation (ATR) measurement	ATR ≥5°	Screening-positive	327/3,248	10.07%
S050 [73]	Yonghong Ren et al. (2021)	2021	Xixia District, Yinchuan, Ningxia Hui Autonomous Region	School-based cross-sectional screening	Primary and junior secondary school students; 9–16 years	936	postural examination + Adams forward-bend test + angle of trunk rotation (ATR) measurement	ATR ≥5°	Screening-positive	118/936	12.61%
S051 [74]	Yi Sun et al. (2021)	2020	Guangzhou, Guangdong Province	School-based cross-sectional screening	Junior and senior secondary and vocational high school students; within the target age range of 6–18 years; inferred from school stage	2,121	postural examination + Adams forward-bend test + angle of trunk rotation (ATR) measurement + spinal mobility test + prone-position test	Screening-positive according to GB/T 16133-2014	Screening-positive	174/2,121	8.20%
S052 [75]	Jiantao Wen et al. (2021)	2020	Yuzhong County, Lanzhou, Gansu Province	School-based cross-sectional screening	Mixed primary, junior secondary, and senior secondary school students; within the target age range of 6–18 years	36,728	postural examination + Adams forward-bend test + angle of trunk rotation (ATR) measurement	ATR ≥5°	Screening-positive	827/36,728	2.25%
S053 [76]	Ling Chen et al. (2022)	2020	Cixi, Ningbo, Zhejiang Province	School health surveillance (cross-sectional)	Mixed primary and secondary school students; within the target age range of 6–18 years (age not directly reported; inferred from school stage)	1,886	student health examination plus spinal curvature screening	Screening-positive according to GB/T 16133-2014	Screening-positive	16/1,886	0.85%
S054 [77]	Xiaosheng Chen et al. (2022)	2020	Shenzhen, Guangdong Province	School-based cross-sectional screening	Mixed primary and secondary school students; within the target age range of 6–18 years	969,235	Adams forward-bend test + angle of trunk rotation (ATR) measurement	ATR ≥5°	Screening-positive	48,587/969,235	5.01%

S055 [78]	Meng Li et al. (2022)	2020	Guangdong Province	School health surveillance (cross-sectional)	Mixed primary and secondary school students; within the target age range of 6–18 years	38,649	postural examination + Adams forward-bend test + angle of trunk rotation (ATR) measurement + spinal mobility test + prone-position test	Screening-positive according to GB/T 16133-2014	Screening-positive	1,440/38,649	3.73%
S056 [79]	Haibin Guo et al. (2022)	2020–2021	Xining and Haidong, Qinghai Province	School-based cross-sectional screening	Mixed primary and junior secondary school students; included 5-year-olds; addressed in sensitivity analysis	3,582	postural examination + Adams forward-bend test + angle of trunk rotation (ATR) measurement	ATR ≥5°	Screening-positive	201/3,582	5.61%
S057 [80]	Peixin Liu et al. (2022)	2020	Zhuhai, Guangdong Province	School-based cross-sectional screening	Mixed primary and secondary school students; within the target age range of 6–18 years	5,080	postural examination + Adams forward-bend test + angle of trunk rotation (ATR) measurement	ATR >5°	Screening-positive	158/5,080	3.11%
S058 [81]	Guanghong Pu et al. (2022)	2021	Dali Bai Autonomous Prefecture, Yunnan Province	School-based cross-sectional screening	Mixed primary and secondary school students; within the target age range of 6–18 years	140,026	postural examination + Adams forward-bend test + angle of trunk rotation (ATR) measurement	ATR ≥5°	Screening-positive	3,190/140,026	2.28%
S059 [82]	Bin Qiao et al. (2022)	2020	Liangzhou District, Wuwei, Gansu Province	School-based cross-sectional screening	Mixed primary and secondary school students; within the target age range of 6–18 years	3,053	postural examination + Adams forward-bend test + angle of trunk rotation (ATR) measurement + spinal mobility test + prone-position test	Screening-positive criterion defined in the original study	Screening-positive	271/3,053	8.88%
S060 [83]	Yanyan Qiu et al. (2022)	2020–2021	Jiangyin, Wuxi, Jiangsu Province	School-based cross-sectional screening	Mixed primary and secondary school students; within the target age range of 6–18 years	18,562	postural examination + angle of trunk rotation (ATR) measurement + radiographic Cobb-angle measurement	Cobb angle ≥10°	Radiographically confirmed	163/18,562	0.88%
S061 [84]	Xinwen Xu et al. (2022)	2020	Gansu Province	School-based cross-sectional screening	Mixed primary and secondary school students; within the target age range of 6–18 years, inferred from school stage	66,996	spinal curvature screening according to GB/T 16133-2014	Screening-positive according to GB/T 16133-2014	Screening-positive	1,281/66,996	1.91%
S062 [85]	Run Yang et al. (2022)	2022	Taiyuan, Shanxi Province	School-based cross-sectional screening	Mixed primary and secondary school students; within the target age range of 6–18 years	22,564	postural examination + Adams forward-bend test + radiographic Cobb-angle measurement	Cobb angle ≥10°	Radiographically confirmed	71/22,564	0.31%
S063 [86]	Jing Zhao et al. (2022)	2021	Inner Mongolia Autonomous Region	School-based cross-sectional screening	Mixed primary and secondary school students; within the target age range of 6–18 years	92,094	spinal curvature screening according to GB/T 16133-2014	Screening-positive according to GB/T 16133-2014	Screening-positive	1,768/92,094	1.92%
S064 [87]	Yue Zhou et al. (2023)	2022	Nantong, Jiangsu Province	School-based cross-sectional screening	Mixed primary and secondary school students; 7–18 years (participants aged 19 years excluded using single-year age strata)	8,872	postural examination + Adams forward-bend test + angle of trunk rotation (ATR) measurement + spinal mobility test + prone-position test	Screening-positive according to GB/T 16133-2014	Screening-positive	352/8,872	3.97%
S065 [88]	Jin Zhou et al. (2023)	2021	Dali Bai Autonomous Prefecture, Yunnan Province	School-based cross-sectional screening	Mixed primary and secondary school students; within the target age range of 6–18 years	139,922	postural examination + Adams forward-bend test + angle of trunk rotation (ATR) measurement	ATR ≥5°	Screening-positive	3,190/139,922	2.28%
S066 [89]	Shenglei Duan et al. (2023)	2022–2023	Shinan District, Qingdao, Shandong Province	School-based cross-sectional screening	Primary school students; within the target age range of 6–18 years	1,601	postural examination + Adams forward-bend test + angle of trunk rotation (ATR) measurement	ATR ≥5°	Screening-positive	81/1,601	5.06%
S067 [90]	Chang Gao et al. (2023)	2021–2022	Tangshan urban area (including Hangu Administrative District), Hebei Province	School-based cross-sectional screening	Mixed primary and junior secondary school students; within the target age range of 6–18 years	85,146	Adams forward-bend test + angle of trunk rotation (ATR) measurement + spinal mobility test + radiographic Cobb-angle measurement	Cobb angle >10°	Radiographically confirmed	2,080/85,146	2.44%
S068 [91]	Sizhe Huang et al. (2023)	2020	Zhongshan, Guangdong Province	School-based cross-sectional screening	Mixed primary and secondary school students; within the target age range of 6–18 years	6,757	Adams forward-bend test + angle of trunk rotation (ATR) measurement	Screening-positive criterion defined in the original study	Screening-positive	135/6,757	2.00%
S069 [92]	Yan Li et al. (2024)	2023	Huaiyin District, Jinan, Shandong Province	School-based cross-sectional screening	Mixed primary and secondary school students; within the target age range of 6–18 years; inferred from school stage	1,717	postural examination + Adams forward-bend test + angle of trunk rotation (ATR) measurement + spinal mobility test	Screening-positive according to GB/T 16133-2014	Screening-positive	96/1,717	5.59%
S070 [93]	Xihai Li et al. (2023)	2021	Fujian Province	School health surveillance (cross-sectional)	Mixed primary and junior secondary school students; within the target age range of 6–18 years	5,926	Adams forward-bend test + angle of trunk rotation (ATR) measurement + electronic device-assisted assessment	ATR ≥5°	Screening-positive	515/5,926	8.69%
S071 [94]	Yingxian Li et al. (2023)	2020	Chongming District, Shanghai	School-based cross-sectional screening	Mixed primary and secondary school students; within the target age range of 6–18 years; inferred from school stage	2,369	Adams forward-bend test + angle of trunk rotation (ATR) measurement	ATR ≥5°	Screening-positive	135/2,369	5.70%
S072 [95]	Xinyue Ma et al. (2023)	2020, 2021, 2022	Inner Mongolia Autonomous Region	Repeated cross-sectional surveys over consecutive years	Mixed primary and secondary school students; within the target age range of 6–18 years; inferred from primary/secondary school stage	533,457	postural examination + Adams forward-bend test + angle of trunk rotation (ATR) measurement + spinal mobility test + prone-position test	Screening-positive according to GB/T 16133-2014	Screening-positive	10,432/533,457	1.96%
S073 [96]	Jingjing Qiao et al. (2023)	2021	Dongcheng District, Beijing	School health surveillance (cross-sectional)	Mixed primary and secondary school students; within the target age range of 6–18 years; inferred from school stage	1,539	postural examination + Adams forward-bend test + angle of trunk rotation (ATR) measurement + spinal mobility test + prone-position test	Screening-positive according to GB/T 16133-2014	Screening-positive	71/1,539	4.61%

S074 [97]	Xianwei Zhang et al. (2023)	2021	Tianjin	School-based cross-sectional screening	Mixed junior and senior secondary school students; 11–16 years	17,629	postural examination + Adams forward-bend test + angle of trunk rotation (ATR) measurement + spinal mobility test + prone-position test	ATR ≥5°	Screening-positive	695/17,629	3.94%
S075 [98]	Fei Wang et al. (2023)	2020	Zhangjiajie, Hunan Province	School-based cross-sectional screening	Mixed primary, junior secondary, and senior secondary school students; not directly reported; participants were primary, junior secondary, and senior secondary school students	4,866	Adams forward-bend test + angle of trunk rotation (ATR) measurement + spinal mobility test + prone-position test	Screening-positive according to GB/T 16133-2014	Screening-positive	126/4,866	2.59%
S076 [99]	Jianwei Wei et al. (2023)	2020–2022	Laoshan District, Qingdao, Shandong Province	School-based cross-sectional screening	Primary and junior secondary school students; within the target age range of 6–18 years	17,520	postural examination + Adams forward-bend test	Cobb angle >10°	Radiographically confirmed	114/17,520	0.65%
S077 [100]	Dan Yu et al. (2023)	2021	Shiyan, Hubei Province	School-based cross-sectional screening	Mixed primary and secondary school students; within the target age range of 6–18 years	1,674	Adams forward-bend test + angle of trunk rotation (ATR) measurement + radiographic Cobb-angle measurement	Cobb angle >10°	Radiographically confirmed	113/1,674	6.75%
S078 [101]	Wei Zhang et al. (2023)	2020–2022	Henan Province	School-based cross-sectional screening	Primary and junior secondary school students; within the target age range of 6–18 years	27,926	postural examination + Adams forward-bend test + radiographic Cobb-angle measurement	Cobb angle ≥10°	Radiographically confirmed	1,067/27,926	3.82%
S079 [102]	Jing Zhao et al. (2023)	2021	Inner Mongolia Autonomous Region	School-based cross-sectional screening	Mixed primary and junior secondary school students; within the target age range of 6–18 years	18,399	physical screening for spinal curvature abnormalities by trained professionals according to GB/T 16133-2014	Postural scoliosis criterion defined in the original study	Screening-positive	368/18,399	2.00%
S080 [103]	Haibin Guo et al. (2023)	2020–2021	Xining and Haidong, Qinghai Province	School-based cross-sectional screening	Mixed primary and junior secondary school students; 10–18 years	1,549	postural examination + Adams forward-bend test + angle of trunk rotation (ATR) measurement	ATR ≥5°	Screening-positive	143/1,549	9.23%
S081 [25]	Jin Huang et al. (2024)	2022	Baiyin, Jinchang, Lanzhou, Linxia Hui Autonomous Prefecture, and Gannan Tibetan Autonomous Prefecture, Gansu Province	School-based cross-sectional screening	Mixed junior and senior secondary school students; within the target age range of 6–18 years	3,044	Adams forward-bend test + angle of trunk rotation (ATR) measurement + radiographic Cobb-angle measurement	Screening-positive criterion defined in the original study	Screening-positive	173/3,044	5.68%
S082 [104]	Qi Huang et al. (2026)	2022	Chongqing	School-based cross-sectional screening	Mixed primary, junior secondary, and senior secondary school students; generally within the target age range of 6–18 years	13,605	postural examination + Adams forward-bend test + angle of trunk rotation (ATR) measurement + spinal mobility test	ATR ≥5°	Screening-positive	124/13,605	0.91%
S083 [105]	Lu Liu et al. (2024)	2021	Shijiazhuang, Hebei Province	School-based cross-sectional screening	Mixed primary and secondary school students; 7–18 years	181,935	Screening: postural examination + Adams forward-bend test + angle of trunk rotation (ATR) measurement Confirmation: radiographic Cobb-angle measurement	Screening: ATR ≥5° Confirmation: Cobb angle >10°	Screening-positive; radiographically confirmed	Screening: 16,676/181,935 Confirmed: 289/181,935	Screening: 9.17% Confirmed: 0.16%
S084 [106]	Jun Song et al. (2024)	2021	Central urban districts of Nanjing, Jiangsu Province	School-/kindergarten-based cross-sectional screening	Mixed preschool and lower-primary children; 6–7 years	764	postural examination + Adams forward-bend test + angle of trunk rotation (ATR) measurement + radiographic Cobb-angle measurement + electronic device-assisted assessment	Cobb angle ≥10°	Radiographically confirmed	51/764	6.68%
S085 [107]	Weidi Sun et al. (2024)	2021	Zhejiang Province	School health surveillance (cross-sectional)	Mixed primary, junior secondary, and senior secondary school students; 11–17 years	21,061	postural examination + Adams forward-bend test	ATR criterion defined in the original study	Screening-positive	1,077/21,061	5.11%
S086 [108]	Ruoyi Gao et al. (2024)	2020, 2021, 2022	Beijing	Repeated cross-sectional surveys over consecutive years	Mixed primary, junior secondary, senior secondary, and vocational high school students; within the target age range of 6–18 years	105,927	spinal curvature screening according to the Technical Guidelines for Prevention and Control of Spinal Curvature Abnormalities in Children and Adolescents	Positive clinical signs	Screening-positive	1,846/105,927	1.74%
S087 [109]	Yan Han et al. (2024)	2022	Minhang District, Shanghai	School-based cross-sectional screening	Mixed primary, junior secondary, and senior secondary school students; included 19-year-olds; sensitivity analysis applied	5,715	postural examination + Adams forward-bend test + angle of trunk rotation (ATR) measurement + spinal mobility test + prone-position test	Screening-positive according to GB/T 16133-2014	Screening-positive	218/5,715	3.81%
S088 [110]	Qin Zhang et al. (2024)	2021	Jiading District, Shanghai	School-based cross-sectional screening	Primary and junior secondary school students; primary and junior secondary school students	1,133	postural examination + Adams forward-bend test + angle of trunk rotation (ATR) measurement + spinal mobility test	ATR ≥5°	Screening-positive	42/1,133	3.71%
S089 [111]	Wei Zhou et al. (2024)	2021	Jiangbei District, Ningbo, Zhejiang Province	School-based cross-sectional screening	Mixed primary, junior secondary, and senior secondary school students; approximately 9–18 years	1,639	postural examination + Adams forward-bend test + angle of trunk rotation (ATR) measurement	Screening-positive criterion defined in the original study	Screening-positive	113/1,639	6.89%
S090 [112]	Liting Chu et al. (2025)	2021, 2022, 2023	16 districts of Shanghai	Longitudinal dynamic cohort	Primary and junior secondary school students; grade 1 through grade 8	19,673	Adams forward-bend test + angle of trunk rotation (ATR) measurement + spinal mobility test	Screening-positive criterion defined in the original study	Screening-positive	223/19,673	1.13%
S091 [113]	Xiaofan Du et al. (2025)	2022–2024	Laoshan District, Qingdao, Shandong Province	Repeated cross-sectional surveys over consecutive years	Primary and junior secondary school students; 8–15 years	57,393	postural examination + Adams forward-bend test + angle of trunk rotation (ATR) measurement	ATR ≥5°	Screening-positive	2,614/57,393	4.55%

S092 [114]	Lihua Jin et al. (2025)	2020–2022	Yunnan Province	School-based cross-sectional screening	Junior and senior secondary school students; 12–17 years	765	angle of trunk rotation (ATR) measurement	ATR ≥5°	Screening-positive	90/765	11.76%
S093 [115]	Xuedong Ma et al. (2024)	2020, 2021, 2022	Jinze Town, Qingpu District, Shanghai	Repeated cross-sectional surveys over consecutive years	Mixed primary and secondary school students; primary and secondary school students	2,021	postural examination + Adams forward-bend test + angle of trunk rotation (ATR) measurement + spinal mobility test	ATR criterion defined in the original study	Screening-positive	76/2,021	3.76%
S094 [116]	Chengzhi Jiang et al. (2024)	2022	Science City area, Mianyang, Sichuan Province	School-based cross-sectional screening	Junior and senior secondary school students; 10–18 years	1,952	postural examination + Adams forward-bend test + angle of trunk rotation (ATR) measurement + radiographic Cobb-angle measurement	Cobb angle ≥10°	Radiographically confirmed	72/1,952	3.69%
S095 [117]	Wei Liang et al. (2024)	2023	Chuzhou, Anhui Province	School-based cross-sectional screening	Mixed primary, junior secondary, and senior secondary school students; 10–18 years	1,768	postural examination + Adams forward-bend test + angle of trunk rotation (ATR) measurement	ATR ≥7°	Screening-positive	131/1,768	7.41%
S096 [118]	Xizhang Liang et al. (2024)	2021–2022	Qingyuan, Guangdong Province	School-based cross-sectional screening	Mixed primary, junior secondary, and senior secondary school students; 6–18 years	665,333	Screening: Adams forward-bend test Confirmation: radiographic Cobb-angle measurement	Screening: positive five-step screening procedure Confirmation: Cobb angle >10°	Screening-positive; radiographically confirmed	Screening: 3,122/665,333 Confirmed: 1,824/665,333	Screening: 0.47% Confirmed: 0.27%
S097 [119]	Qingxin Ren et al. (2024)	2021	Chongchuan District and Qidong, Nantong, Jiangsu Province	School-based cross-sectional screening	Primary and junior secondary school students; primary and junior secondary school students	3,189	Adams forward-bend test + angle of trunk rotation (ATR) measurement + spinal mobility test + prone-position test	Screening-positive according to GB/T 16133-2014	Screening-positive	77/3,189	2.41%
S098 [120]	Xianwei Zhang et al. (2024)	2023	16 districts of Tianjin	School-based cross-sectional screening	Mixed primary, junior secondary, and senior secondary school students; primary and secondary school students	41,654	postural examination + Adams forward-bend test + angle of trunk rotation (ATR) measurement + spinal mobility test + prone-position test	ATR ≥5°	Screening-positive	1,540/41,654	3.70%
S099 [121]	Dandan Wang et al. (2024)	2023	Nanhu District, Jiaxing, Zhejiang Province	School-based cross-sectional screening	Mixed primary, junior secondary, and senior secondary school students; upper-primary through senior secondary school students	1,854	postural examination + Adams forward-bend test + angle of trunk rotation (ATR) measurement + spinal mobility test + prone-position test	Screening-positive according to GB/T 16133-2014	Screening-positive	103/1,854	5.56%
S100 [122]	Kun Liu et al. (2024)	2022	Hanyang District, Wuhan, Hubei Province	School-based cross-sectional screening	Primary and junior secondary school students; 10–16 years	2,274	postural examination + Adams forward-bend test + angle of trunk rotation (ATR) measurement + radiographic Cobb-angle measurement	Cobb angle ≥10°	Radiographically confirmed	61/2,274	2.68%
S101 [123]	Lijuan Zhao et al. (2025)	2021	Four urban/county districts of Hangzhou, Zhejiang Province	School-based cross-sectional screening	Mixed primary, junior secondary, and senior secondary school students; upper-primary through senior secondary school students	5,652	postural examination + Adams forward-bend test + angle of trunk rotation (ATR) measurement + spinal mobility test	ATR ≥5°	Screening-positive	125/5,652	2.21%
S102 [124]	Shuaiqi Zhu et al. (2025)	2023	Eight counties/cities in Linxia Hui Autonomous Prefecture, Gansu Province	School-based cross-sectional screening	Primary and junior secondary school students; approximately 10–16 years	27,532	angle of trunk rotation (ATR) measurement + spinal mobility test + electronic device-assisted assessment	ATR ≥5°	Screening-positive	624/27,532	2.27%
S103 [125]	Siyu Chen et al. (2025)	2023	Yangzhou, Jiangsu Province	School health surveillance (cross-sectional)	Mixed primary, junior secondary, and senior secondary school students; 6–18 years	3,854	Adams forward-bend test + angle of trunk rotation (ATR) measurement	ATR ≥5°	Screening-positive	109/3,854	2.83%

Supplementary Table S2B. Screening-program classification and radiographic follow-up (n = 103)

Study ID	Screening program setting	Classification basis	Classification certainty	Radiographic referral/follow-up data	Reported/calculated completion	Radiographic follow-up status
S001 [26]	Investigator-initiated/local academic study	Full-text verification: investigator-initiated/local cross-sectional screening study.	Clear	NA	NA	NA
S002 [27]	Routine/official public-health screening	Study design explicitly reported as school health surveillance.	Clear	NA	NA	NA
S003 [28]	Investigator-initiated/local academic study	Full-text verification: investigator-initiated/local cross-sectional screening study.	Clear	NA	NA	NA
S004 [29]	Investigator-initiated/local academic study	Full-text verification: investigator-initiated/local cross-sectional screening study.	Clear	NA	NA	NA
S005 [30]	Investigator-initiated/local academic study	Full-text verification: investigator-initiated/local cross-sectional screening study.	Clear	NA	NA	NA
S006 [31]	Investigator-initiated/local academic study	Full-text verification: investigator-initiated/local cross-sectional screening study.	Clear	NA	NA	NA
S007 [32]	Investigator-initiated/local academic study	Full-text verification: investigator-initiated/local cross-sectional screening study.	Clear	NA	NA	NA
S008 [33]	Investigator-initiated/local academic study	Full-text verification: investigator-initiated/local cross-sectional screening study.	Clear	NA	NA	NA
S009 [34]	Routine/official public-health screening	Study design explicitly reported as school health surveillance.	Clear	NA	NA	NA
S010 [35]	Investigator-initiated/local academic study	Full-text verification: investigator-initiated/local cross-sectional screening study.	Clear	NA	NA	NA
S011 [36]	Routine/official public-health screening	Full-text verification: routine/official student health or public-health screening program.	Clear	NA	NA	NA
S012 [37]	Investigator-initiated/local academic study	Full-text verification: investigator-initiated/local cross-sectional screening study.	Clear	NA	NA	NA
S013 [38]	Unclear	Full text did not provide sufficient program or organizational information for reliable classification.	Unclear	NA	NA	NA
S014 [39]	Investigator-initiated/local academic study	Full-text verification: investigator-initiated/local cross-sectional screening study.	Clear	NA	NA	NA
S015 [40]	Investigator-initiated/local academic study	Full-text verification: investigator-initiated/local cross-sectional screening study.	Clear	NA	NA	NA
S016 [41]	Investigator-initiated/local academic study	Full-text verification: investigator-initiated/local cross-sectional screening study.	Clear	NA	NA	NA
S017 [42]	Routine/official public-health screening	Full-text verification: routine/official student health or public-health screening program.	Clear	NA	NA	NA
S018 [43]	Routine/official public-health screening	Study design explicitly reported as school health surveillance.	Clear	NA	NA	NA
S019 [44]	Investigator-initiated/local academic study	Full-text verification: investigator-initiated/local cross-sectional screening study.	Clear	NA	NA	NA
S020 [45]	Investigator-initiated/local academic study	Full-text verification: investigator-initiated/local cross-sectional screening study.	Clear	2,401 suspected AIS; 2,381 completed X-ray; 20 refused radiography.	99.2% (2,381/2,401)	Documented incomplete
S021 [46]	Routine/official public-health screening	Full-text verification: routine/official student health or public-health screening program.	Clear	NA	NA	NA
S022 [126]	Investigator-initiated/local academic study	Full-text verification: investigator-initiated/local cross-sectional screening study.	Clear	NR	NR	Not reported
S023 [47]	Routine/official public-health screening	Full-text verification: routine/official student health or public-health screening program.	Clear	NA	NA	NA
S024 [48]	Routine/official public-health screening	Full-text verification: routine/official student health or public-health screening program.	Clear	NA	NA	NA
S025 [24]	Routine/official public-health screening	Full-text verification: routine/official student health or public-health screening program.	Clear	NA	NA	NA
S026 [49]	Investigator-initiated/local academic study	Full-text verification: investigator-initiated/local cross-sectional screening study.	Clear	235 screen-positive; 215 attended hospital re-screening; X-ray completion not separately reported.	91.4% hospital re-screening (215/235); X-ray completion NR	Partial pathway information; strict completion rate unavailable
S027 [50]	Unclear	Full text did not provide sufficient program or organizational information for reliable classification.	Unclear	NA	NA	NA
S028 [51]	Investigator-initiated/local academic study	Full-text verification: investigator-initiated/local cross-sectional screening study.	Clear	NR	NR	Not reported
S029 [52]	Routine/official public-health screening	Full-text verification: routine/official student health or public-health screening program.	Clear	NA	NA	NA

S030 [53]	Investigator-initiated/local academic study	Full-text verification: investigator-initiated/local cross-sectional screening study.	Clear	Second-screen positive students were advised to undergo third-stage radiography; exact n completing X-ray not reported.	82.8% third-stage attendance	Documented incomplete
S031 [54]	Investigator-initiated/local academic study	Full-text verification: investigator-initiated/local cross-sectional screening study.	Clear	19 initial positives; 17 second-screen positives; X-ray completion n not reported; 13 confirmed.	NR	Not reported
S032 [55]	Unclear	Full text did not provide sufficient program or organizational information for reliable classification.	Unclear	ATR >=5 degrees triggered X-ray assessment; referral-eligible and X-ray-completed counts not separately reported.	NR	Not reported
S033 [56]	Routine/official public-health screening	Full-text verification: routine/official student health or public-health screening program.	Clear	NA	NA	NA
S034 [57]	Investigator-initiated/local academic study	Full-text verification: investigator-initiated/local cross-sectional screening study.	Clear	Clinical/ATR-positive participants underwent imaging; referral-eligible and X-ray-completed counts not separately reported.	NR	Not reported
S035 [58]	Routine/official public-health screening	Full-text verification: routine/official student health or public-health screening program.	Clear	NA	NA	NA
S036 [59]	Investigator-initiated/local academic study	Full-text verification: investigator-initiated/local cross-sectional screening study.	Clear	Sequential screening included X-ray examination; referral-eligible and X-ray-completed counts not separately reported.	NR	Not separately reported
S037 [60]	Investigator-initiated/local academic study	Full-text verification: investigator-initiated/local cross-sectional screening study.	Clear	45 screen-positive participants; all 45 completed X-ray; 22 confirmed.	100% (45/45)	Documented complete
S038 [61]	Investigator-initiated/local academic study	Full-text verification: investigator-initiated/local cross-sectional screening study.	Clear	NA	NA	NA
S039 [62]	Routine/official public-health screening	Study design explicitly reported as school health surveillance.	Clear	NA	NA	NA
S040 [63]	Investigator-initiated/local academic study	Full-text verification: investigator-initiated/local cross-sectional screening study.	Clear	140 initial screen-positive; second-screen positive and X-ray-completed counts not separately reported; 88 confirmed.	NR	Not reported
S041 [64]	Investigator-initiated/local academic study	Full-text verification: investigator-initiated/local cross-sectional screening study.	Clear	NA	NA	NA
S042 [65]	Unclear	Full text did not provide sufficient program or organizational information for reliable classification.	Unclear	NA	NA	NA
S043 [66]	Investigator-initiated/local academic study	Full-text verification: investigator-initiated/local cross-sectional screening study.	Clear	NA	NA	NA
S044 [67]	Investigator-initiated/local academic study	Full-text verification: investigator-initiated/local cross-sectional screening study.	Clear	Abnormal screening results were referred for full-spine X-ray; referral-eligible and X-ray-completed counts not reported.	NR	Not reported
S045 [68]	Investigator-initiated/local academic study	Full-text verification: investigator-initiated/local cross-sectional screening study.	Clear	NA	NA	NA
S046 [69]	Routine/official public-health screening	Full-text verification: routine/official student health or public-health screening program.	Clear	NA	NA	NA
S047 [70]	Investigator-initiated/local academic study	Full-text verification: investigator-initiated/local cross-sectional screening study.	Clear	NA	NA	NA
S048 [71]	Routine/official public-health screening	Full-text verification: routine/official student health or public-health screening program.	Clear	NA	NA	NA
S049 [72]	Investigator-initiated/local academic study	Full-text verification: investigator-initiated/local cross-sectional screening study.	Clear	NA	NA	NA
S050 [73]	Investigator-initiated/local academic study	Full-text verification: investigator-initiated/local cross-sectional screening study.	Clear	NA	NA	NA
S051 [74]	Routine/official public-health screening	Full-text verification: routine/official student health or public-health screening program.	Clear	NA	NA	NA
S052 [75]	Investigator-initiated/local academic study	Full-text verification: investigator-initiated/local cross-sectional screening study.	Clear	NA	NA	NA
S053 [76]	Routine/official public-health screening	Study design explicitly reported as school health surveillance.	Clear	NA	NA	NA
S054 [77]	Routine/official public-health screening	Full-text verification: routine/official student health or public-health screening program.	Clear	NA	NA	NA
S055 [78]	Routine/official public-health screening	Study design explicitly reported as school health surveillance.	Clear	NA	NA	NA
S056 [79]	Investigator-initiated/local academic study	Full-text verification: investigator-initiated/local cross-sectional screening study.	Clear	NA	NA	NA
S057 [80]	Routine/official public-health screening	Full-text verification: routine/official student health or public-health screening program.	Clear	NA	NA	NA
S058 [81]	Investigator-initiated/local academic study	Full-text verification: investigator-initiated/local cross-sectional screening study.	Clear	NA	NA	NA
S059 [82]	Routine/official public-health screening	Full-text verification: routine/official student health or public-health screening program.	Clear	NA	NA	NA
S060 [83]	Investigator-initiated/local academic study	Full-text verification: investigator-initiated/local cross-sectional screening study.	Clear	273 physical-exam positive; 201 scoliometer positive; X-ray-completed count not separately reported; 163 confirmed.	NR	Not separately reported

S061 [84]	Routine/official public-health screening	Full-text verification: routine/official student health or public-health screening program.	Clear	NA	NA	NA
S062 [85]	Investigator-initiated/local academic study	Full-text verification: investigator-initiated/local cross-sectional screening study.	Clear	826 suspected scoliosis; participants with positive screening were referred for full-spine X-ray; X-ray-completed count not reported.	NR	Not reported
S063 [86]	Routine/official public-health screening	Full-text verification: routine/official student health or public-health screening program.	Clear	NA	NA	NA
S064 [87]	Routine/official public-health screening	Full-text verification: routine/official student health or public-health screening program.	Clear	NA	NA	NA
S065 [88]	Investigator-initiated/local academic study	Full-text verification: investigator-initiated/local cross-sectional screening study.	Clear	NA	NA	NA
S066 [89]	Investigator-initiated/local academic study	Full-text verification: investigator-initiated/local cross-sectional screening study.	Clear	NA	NA	NA
S067 [90]	Routine/official public-health screening	Full-text verification: routine/official student health or public-health screening program.	Clear	17,233 initial spinal abnormalities; 4,107 attended hospital and completed full-spine X-ray; 2,080 confirmed.	23.8% (4,107/17,233)	Documented incomplete
S068 [91]	Routine/official public-health screening	Full-text verification: routine/official student health or public-health screening program.	Clear	NA	NA	NA
S069 [92]	Routine/official public-health screening	Full-text verification: routine/official student health or public-health screening program.	Clear	NA	NA	NA
S070 [93]	Routine/official public-health screening	Study design explicitly reported as school health surveillance.	Clear	NA	NA	NA
S071 [94]	Routine/official public-health screening	Full-text verification: routine/official student health or public-health screening program.	Clear	NA	NA	NA
S072 [95]	Routine/official public-health screening	Full-text verification: routine/official student health monitoring across survey years.	Clear	NA	NA	NA
S073 [96]	Routine/official public-health screening	Study design explicitly reported as school health surveillance.	Clear	NA	NA	NA
S074 [97]	Routine/official public-health screening	Full-text verification: routine/official student health or public-health screening program.	Clear	NA	NA	NA
S075 [98]	Investigator-initiated/local academic study	Full-text verification: investigator-initiated/local cross-sectional screening study.	Clear	NA	NA	NA
S076 [99]	Investigator-initiated/local academic study	Full-text verification: investigator-initiated/local cross-sectional screening study.	Clear	935 first-screen suspected; 432 attended hospital and completed full-spine X-ray; 114 confirmed.	46.2% (432/935)	Documented incomplete
S077 [100]	Investigator-initiated/local academic study	Full-text verification: investigator-initiated/local cross-sectional screening study.	Clear	Abnormal screening results were referred for full-spine X-ray; referral-eligible and X-ray-completed counts not reported.	NR	Not reported
S078 [101]	Investigator-initiated/local academic study	Full-text verification: investigator-initiated/local cross-sectional screening study.	Clear	Screen-positive participants were scheduled for full-spine X-ray; referral-eligible and X-ray-completed counts not reported.	NR	Not reported
S079 [102]	Routine/official public-health screening	Full-text verification: routine/official student health or public-health screening program.	Clear	NA	NA	NA
S080 [103]	Investigator-initiated/local academic study	Full-text verification: investigator-initiated/local cross-sectional screening study.	Clear	NA	NA	NA
S081 [25]	Investigator-initiated/local academic study	Full-text verification: investigator-initiated/local cross-sectional screening study.	Clear	NA	NA	NA
S082 [104]	Investigator-initiated/local academic study	Full-text verification: investigator-initiated/local cross-sectional screening study.	Clear	NA	NA	NA
S083 [105]	Routine/official public-health screening	Full-text verification: routine/official student health or public-health screening program.	Clear	16,676 first-screen positive; 2,054 completed hospital re-screening; 1,256 second-screen positive; 705 underwent imaging; 289 confirmed.	Hospital re-screening 12.3% (2,054/16,676); imaging among second-screen positives 56.1% (705/1,256)	Documented incomplete
S084 [106]	Investigator-initiated/local academic study	Full-text verification: investigator-initiated/local cross-sectional screening study.	Clear	Study-wide pathway: 173 screen-positive and 134 X-ray-confirmed; the included meta-analysis effect used the age 6-7-year subgroup, for which completion was not separately reported.	Study-wide PPV 77.5% (134/173); age-specific completion NR	Not determinable for included subgroup
S085 [107]	Routine/official public-health screening	Study design explicitly reported as school health surveillance.	Clear	NA	NA	NA
S086 [108]	Routine/official public-health screening	Full-text verification: routine/official student health monitoring across survey years.	Clear	NA	NA	NA
S087 [109]	Unclear	Full text did not provide sufficient program or organizational information for reliable classification.	Unclear	NA	NA	NA
S088 [110]	Routine/official public-health screening	Full-text verification: routine/official student health or public-health screening program.	Clear	NA	NA	NA
S089 [111]	Routine/official public-health screening	Full-text verification: routine/official student health or public-health screening program.	Clear	NA	NA	NA

S090 [112]	Routine/official public-health screening	Full-text verification: routine/official student health or public-health screening program.	Clear	NA	NA	NA
S091 [113]	Routine/official public-health screening	Full-text verification: routine/official student health monitoring across survey years.	Clear	NA	NA	NA
S092 [114]	Investigator-initiated/local academic study	Full-text verification: investigator-initiated/local cross-sectional screening study.	Clear	NA	NA	NA
S093 [115]	Routine/official public-health screening	Full-text verification: routine/official student health monitoring across survey years.	Clear	NA	NA	NA
S094 [116]	Investigator-initiated/local academic study	Full-text verification: investigator-initiated/local cross-sectional screening study.	Clear	ATR >=5 degrees or positive forward-bend examination triggered full-spine X-ray; referral-eligible and X-ray-completed counts not reported.	NR	Not reported
S095 [117]	Investigator-initiated/local academic study	Full-text verification: investigator-initiated/local cross-sectional screening study.	Clear	NA	NA	NA
S096 [118]	Investigator-initiated/local academic study	Full-text verification: investigator-initiated/local cross-sectional screening study.	Clear	3,122 five-step screening abnormalities; 2,891 underwent X-ray; formal ATR >=7 degrees referral denominator not separately reported; 1,824 confirmed.	92.6% relative to initial abnormalities (2,891/3,122); formal referral completion NR	Partial pathway information; strict completion rate unavailable
S097 [119]	Routine/official public-health screening	Full-text verification: routine/official student health or public-health screening program.	Clear	NA	NA	NA
S098 [120]	Routine/official public-health screening	Full-text verification: routine/official student health or public-health screening program.	Clear	NA	NA	NA
S099 [121]	Routine/official public-health screening	Full-text verification: routine/official student health or public-health screening program.	Clear	NA	NA	NA
S100 [122]	Investigator-initiated/local academic study	Full-text verification: investigator-initiated/local cross-sectional screening study.	Clear	Second-screen suspected scoliosis cases underwent radiography; referral-eligible and X-ray-completed counts not reported; 61 confirmed.	NR	Not reported
S101 [123]	Investigator-initiated/local academic study	Full-text verification: investigator-initiated/local cross-sectional screening study.	Clear	NA	NA	NA
S102 [124]	Investigator-initiated/local academic study	Full-text verification: investigator-initiated/local cross-sectional screening study.	Clear	NA	NA	NA
S103 [125]	Routine/official public-health screening	Yangzhou 2023 student common-disease surveillance; implemented under the 2022 Jiangsu student common-disease monitoring and intervention program; CDC surveillance data.	Clear	NA	NA	NA

Note: NA, not applicable (study did not contribute a radiographically confirmed outcome); NR, not reported. Screening program setting was classified as routine/official public-health screening, investigator-initiated/local academic study, or unclear. For the 23 studies contributing radiographically confirmed prevalence, radiographic follow-up was classified as documented complete (n = 1), explicitly documented incomplete (n = 5), partial pathway information without a strict radiographic completion rate (n = 2), or not determinable from the published report (n = 15). Referral and follow-up information was extracted from the original reports where available, and completion percentages were calculated only when an explicit numerator and denominator were available.

Etiological classification used for descriptive reporting: 16 studies specifically targeted adolescent idiopathic scoliosis, idiopathic scoliosis, or potential AIS in their screening or outcome definitions (S003, S012, S020, S024, S032, S034, S041, S043, S050, S057, S067, S068, S083, S091, S092, and S096); two studies explicitly differentiated idiopathic from non-idiopathic scoliosis (S010 and S037); the remaining 85 studies did not clearly specify an etiological subtype.

Supplementary Table S3. Operational Criteria for the JBI Critical Appraisal Checklist for Studies Reporting Prevalence Data

JBI item	Assessment domain	Operational criterion used in this review
Q1	Sampling frame	Yes: the sampling frame reasonably represented the target population defined by the study. No: the frame was clearly restricted or inappropriate for the stated target population. Unclear: insufficient information was available to judge representativeness.
Q2	Sampling method	Yes: participants were selected using an appropriate probability-based method (e.g., random, stratified, cluster sampling) or a census/near-census approach. No: convenience or other clearly non-probability selection was used without justification. Unclear: the selection process was insufficiently described.
Q3	Sample size adequacy	Yes: the achieved sample size was adequate for prevalence estimation. This included studies that met a reported sample-size calculation, used a census/near-census design, or, when no a priori calculation was reported, had an achieved sample size that was considered adequate for the expected prevalence and precision of the estimate under the JBI prevalence-study framework. No: the achieved sample size was considered insufficient for estimating prevalence with acceptable precision and there was no census/near-census design or other clear justification for adequacy. Unclear: the achieved sample size or relevant population information was insufficient to judge adequacy. Failure to report an a priori sample-size calculation alone was not grounds for an Unclear rating.
Q4	Participants and setting	Yes: the study population and setting were described in sufficient detail, including location, age or school stage, study period, and relevant population characteristics. No: essential descriptive information was clearly absent. Unclear: reporting was insufficient to determine adequacy.
Q5	Coverage of the identified sample	Yes: the analysis adequately covered the identified sample, and exclusions, missing data, or losses were minimal or appropriately accounted for. No: substantial or systematic loss/exclusion could affect the prevalence estimate and was not adequately handled. Unclear: participant flow or coverage was insufficiently reported.
Q6	Validity of condition identification	Yes: screening positivity or radiographic scoliosis was identified using clearly defined methods and thresholds appropriate to the reported outcome (e.g., standardized school screening procedures, ATR criteria, or radiographic Cobb-angle criteria). No: the outcome definition or identification method was clearly inappropriate or invalid. Unclear: the method or threshold was insufficiently described.
Q7	Standardization and reliability of measurement	Yes: measurements were applied consistently using standardized procedures, with evidence of training, calibration, repeat assessment, quality control, or other reliability safeguards where relevant. No: clear evidence indicated non-standard or unreliable measurement. Unclear: reporting was insufficient to judge consistency or reliability.
Q8	Statistical analysis	Yes: numerators, denominators, and statistical methods were appropriate for the study design and reported outcome, with the sampling design accounted for when required. No: important inconsistencies, inappropriate denominators, or clearly unsuitable analyses were present. Unclear: statistical reporting was insufficient for judgment.
Q9	Response rate and management of nonresponse	Yes: response/completion was adequate, or nonresponse/loss to follow-up was reported and appropriately addressed. No: substantial nonresponse or loss to follow-up was likely to bias the estimate and was not adequately managed. Unclear: the response or completion process was not sufficiently reported. For radiographic outcomes, referral and verification completion were considered when relevant to the analyzed outcome.

Note: Each JBI item was rated as Yes, No, Unclear, or Not applicable. The criteria were applied at the independent-study level. JBI item 3 was reassessed on the basis of achieved sample-size adequacy for prevalence estimation rather than the presence or absence of a reported a priori sample-size calculation. JBI does not prescribe a numerical total score or a universal overall quality category; therefore, item-level judgments were retained for interpretation.

Supplementary Figure S1. Leave-one-study-out sensitivity analyses: (A) screening-positive rate; (B) Cobb angle-confirmed prevalence.

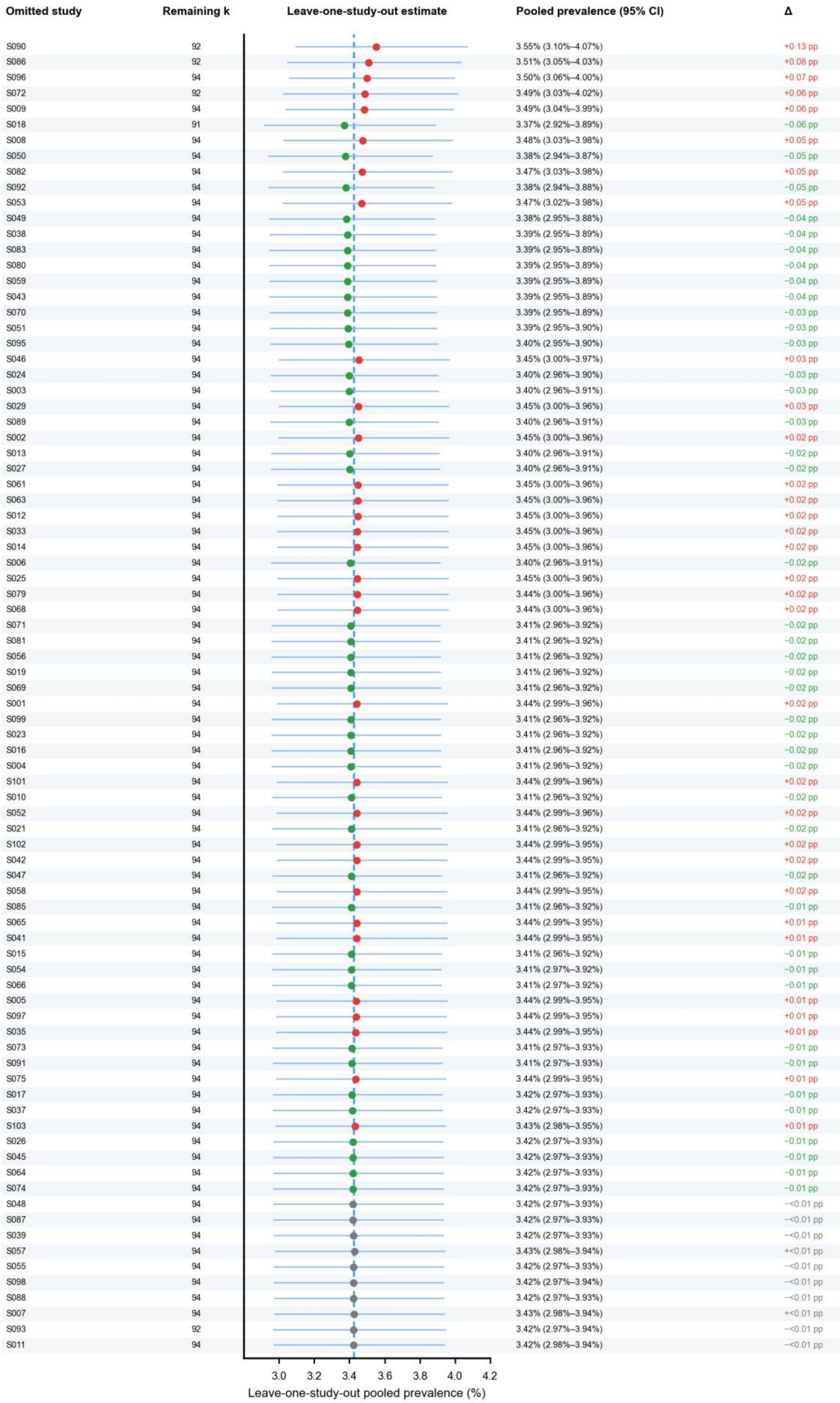

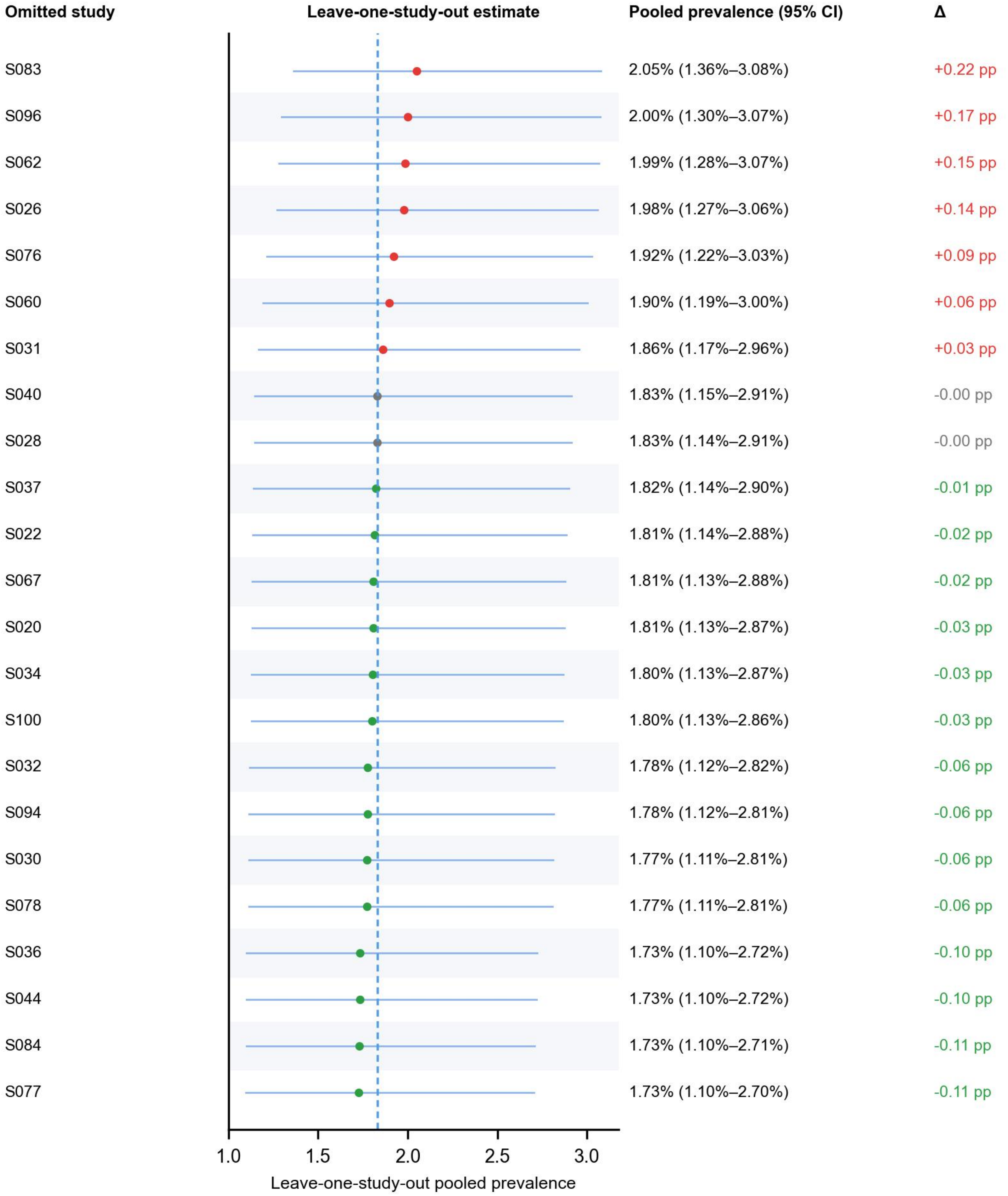
Omitted study
Leave-one-study-out estimate
Pooled prevalence (95% CI)
Δ
S083
2.05% (1.36%–3.08%)
+0.22 pp
S096
2.00% (1.30%–3.07%)
+0.17 pp
S062
1.99% (1.28%–3.07%)
+0.15 pp
S026
1.98% (1.27%–3.06%)
+0.14 pp
S076
1.92% (1.22%–3.03%)
+0.09 pp
S060
1.90% (1.19%–3.00%)
+0.06 pp
S031
1.86% (1.17%–2.96%)
+0.03 pp
S040
1.83% (1.15%–2.91%)
-0.00 pp
S028
1.83% (1.14%–2.91%)
-0.00 pp
S037
1.82% (1.14%–2.90%)
-0.01 pp
S022
1.81% (1.14%–2.88%)
-0.02 pp
S067
1.81% (1.13%–2.88%)
-0.02 pp
S020
1.81% (1.13%–2.87%)
-0.03 pp
S034
1.80% (1.13%–2.87%)
-0.03 pp
S100
1.80% (1.13%–2.86%)
-0.03 pp
S032
1.78% (1.12%–2.82%)
-0.06 pp
S094
1.78% (1.12%–2.81%)
-0.06 pp
S030
1.77% (1.11%–2.81%)
-0.06 pp
S078
1.77% (1.11%–2.81%)
-0.06 pp
S036
1.73% (1.10%–2.72%)
-0.10 pp
S044
1.73% (1.10%–2.72%)
-0.10 pp
S084
1.73% (1.10%–2.71%)
-0.11 pp
S077
1.73% (1.10%–2.70%)
-0.11 pp
1.0
1.5
2.0
2.5
3.0
Leave-one-study-out pooled prevalence

Supplementary Figure S2. Univariable meta-regression for the screening-positive rate: (S2.1) investigation year; (S2.2) ln(sample size); (S2.3) region (eastern China as reference); (S2.4) screening framework classification.

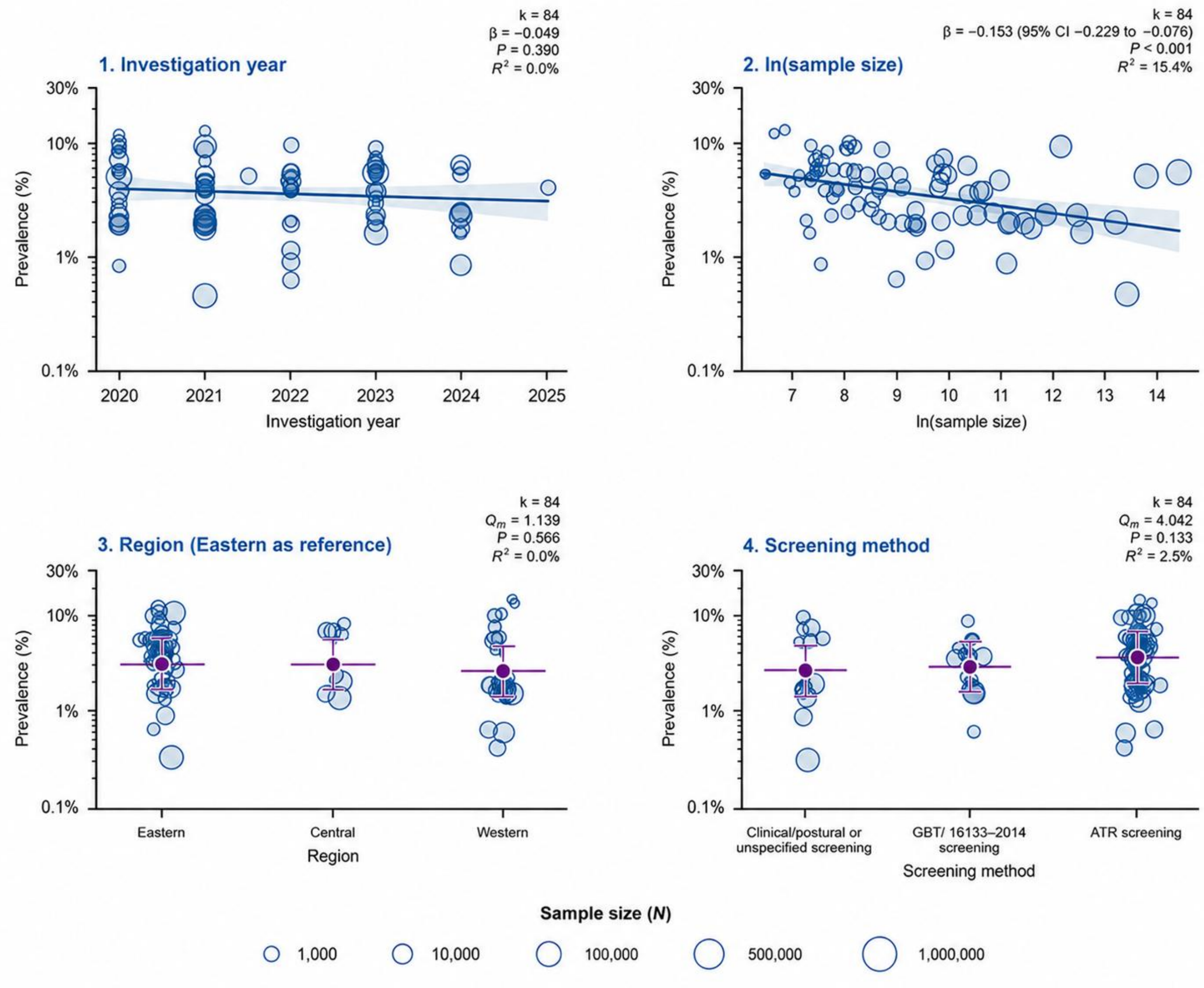

Supplementary Figure S3. Univariable meta-regression for Cobb angle-confirmed prevalence: (S3.1) investigation year; (S3.2) ln (sample size); (S3.3) region (eastern China as reference).

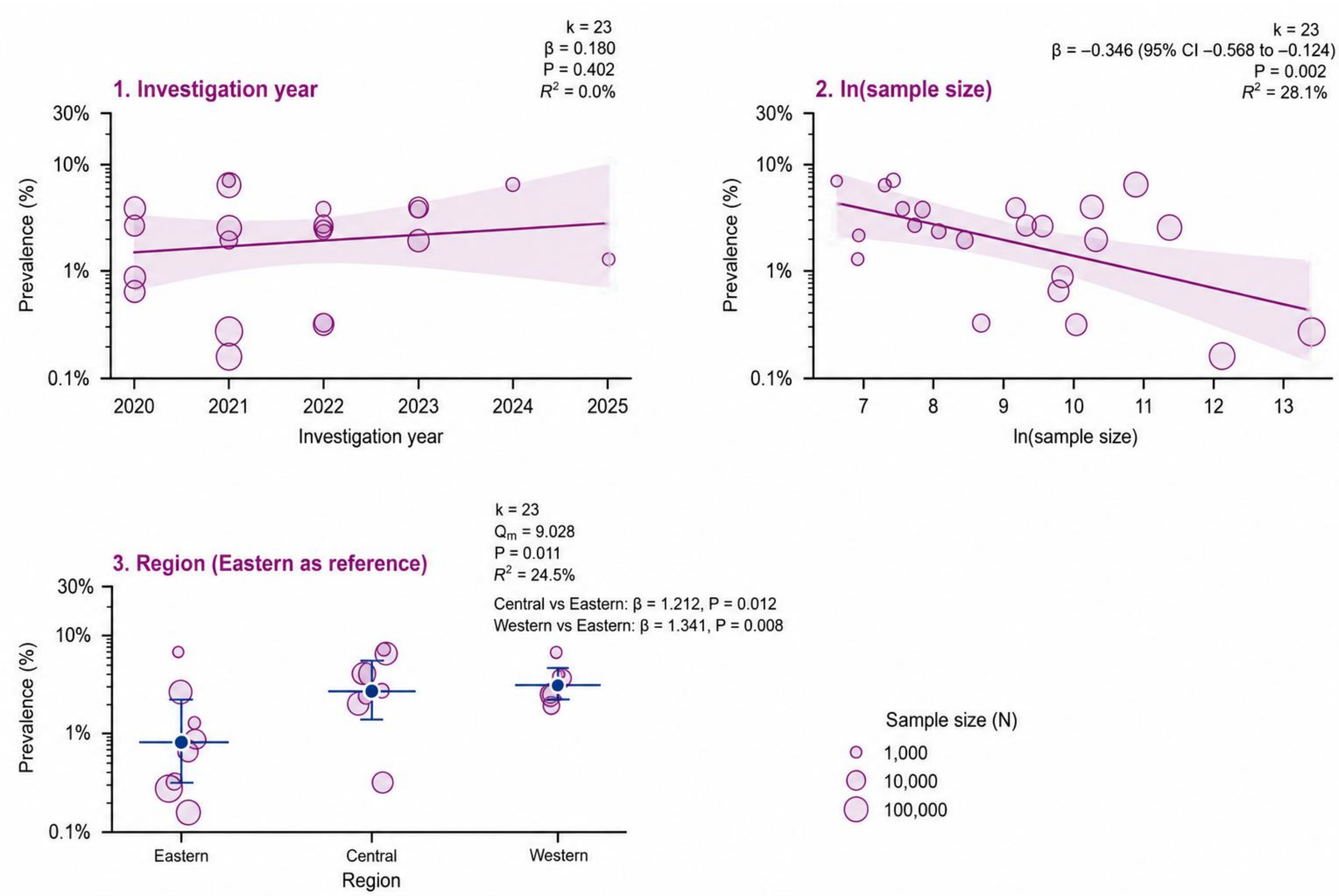

Supplement: Supplementary file 2 [file Datasheet2.pdf]
